# Cellular and Molecular Mechanisms Modulated by Genistein in Cancer

**DOI:** 10.3390/ijms26031114

**Published:** 2025-01-27

**Authors:** Valeria Naponelli, Annamaria Piscazzi, Domenica Mangieri

**Affiliations:** 1 Department of Medicine and Surgery, University of Parma, Plesso Biotecnologico Integrato, Via Volturno 39, 43126 Parma, Italy; 2 Department of Clinical and Experimental Medicine, University of Foggia, Via Pinto 1, 71122 Foggia, Italy

**Keywords:** flavonoid, phytoestrogen, angiogenesis, epithelial–mesenchymal transition, cancer stem cells, cell cycle, programmed cell death

## Abstract

Genistein (4′,5,7-trihydroxyisoflavone) is a phytoestrogen belonging to a subclass of natural flavonoids that exhibits a wide range of pharmacological functions, including antioxidant and anti-inflammatory properties. These characteristics make genistein a valuable phytochemical compound for the prevention and/or treatment of cancer. Genistein effectively inhibits tumor growth and dissemination by modulating key cellular mechanisms. This includes the suppression of angiogenesis, the inhibition of epithelial–mesenchymal transition, and the regulation of cancer stem cell proliferation. These effects are mediated through pivotal signaling pathways such as JAK/STAT, PI3K/Akt/mTOR, MAPK/ERK, NF-κB, and Wnt/β-catenin. Moreover, genistein interferes with the function of specific cyclin/CDK complexes and modulates the activation of Bcl-2/Bax and caspases, playing a critical role in halting tumor cell division and promoting apoptosis. The aim of this review is to discuss in detail the key cellular and molecular mechanisms underlying the pleiotropic anticancer effects of this flavonoid.

## 1. Introduction

Cancer encompasses a diverse group of complex and dynamic diseases, defined by distinct characteristics such as uncontrolled cell proliferation, resistance to apoptosis, epigenetic reprogramming, immune evasion, and phenotypic plasticity [1,2]. Despite their limitations, including severe cytotoxicity, and the development of multidrug resistance, the most employed anticancer and antimetastatic treatments include chemotherapy, radiotherapy, surgery, and immunotherapy, often administered in combination [3]. Furthermore, as one of the leading causes of death worldwide, cancer highlights the need for effective therapeutic approaches, with chemoprevention emerging as one of the most promising strategies [4,5,6]. In this context, several members of the naturally occurring flavonoids, already known for their multiple health benefits, also show efficacy in chemoprevention [7,8,9,10]. Genistein (4′,5,7-trihydroxyisoflavone, a member of the flavonoid family) is a phytoestrogen also acting as a potent inhibitor of tyrosine kinases [11] that has already been successfully tested in the management of breast and prostate cancers [12,13,14,15,16]. Further experimental evidence has demonstrated genistein’s ability to influence a broad spectrum of cell signaling pathways, thus interfering with the progression of several other types of cancer [17,18]. Building on these premises, this review explores the most significant findings on the anticancer properties of genistein, with a focus on its capacity to modulate key molecular pathways involved in angiogenesis, tumor cell migration and invasion, epithelial–mesenchymal transition (EMT), maintenance of cancer stem cells (CSCs), cell cycle arrest, and the induction of tumor cell death.

## 2. Chemistry

**Figure 1:**
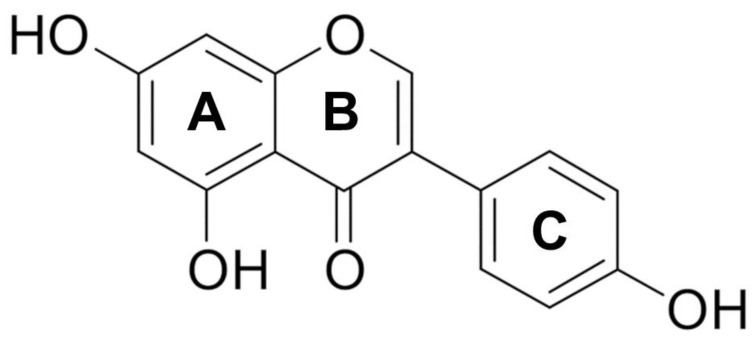
Chemical structure of genistein (5,7-dihydroxy-3-(4-hydroxyphenyl)-chromen-4-one). It is formed by two aromatic benzene rings (A and C) and a non-aromatic heterocyclic pyran ring (B), and the substituents at positions 4′, 5, and 7 of rings A and B are hydroxyl groups.

Genistein (5,7-dihydroxy-3-(4-hydroxyphenyl)-chromen-4-one) is a naturally occurring isoflavone in soy products [19]. Isoflavones are members of the flavonoid family, which comprises over 5000 compounds [15]. The aglycone form of isoflavones is the biologically active form. The biologically active isoflavone is the aglycosylated form obtained after food processing of genistin, found in natural sources [19,20,21]. Knowing the chemical structure of genistein helps to understand its biological activity. Genistein is a 4′,5,7-trihydroxyisoflavone (C15H10O5) with a low molecular weight of 270.24. It is a light-sensitive plant secondary metabolite formed by two aromatic benzene rings (A and C) and a non-aromatic heterocyclic pyran ring (B) (3-phenylchromen-4-one backbone), and the substituents at positions 4′, 5, and 7 of rings A and B are hydroxyl groups (Figure 1) [22]. These phenols give this class of compounds significant antioxidant activity. Moreover, genistein is poorly soluble in water and is soluble in acetone, ethanol, and other polar solvents [21]. Due to its structural similarity to human endogenous estrogen, namely 17β-estradiol (E2), genistein can compete with it and bind to estrogen receptors (ERs) α and β through the two hydroxyl groups located on carbons 4 and 7 [21,23]. Moreover, genistein is a recognized inhibitor of protein kinases [15,24]. This activity is thought to be due to the C4′ phenolic group of this phytoestrogen, which is structurally similar to the phosphoacceptor group of tyrosine [15].

## 3. Sources

Genistein is an isoflavone abundantly found in the roots and seeds of plants belonging to the Fabaceae (Leguminosae) family [25]. The phytoestrogen is abundant in soy foods, soy drinks, and soybeans (Table 1) [21,26]. The genistein content of mature soybeans ranges from 5.6 to 276 mg/100 g, with an average of 81 mg/100 g [26,27]. These differences in genistein content of soybeans are not influenced by the soil cultivation system [28] whereas the technological tillage of soybeans has a significant effect on this parameter. Genistein concentrations are significantly higher in fermented soy products (e.g., miso, tempeh, sufu, natto, soy sauce) compared to unfermented soy and soy-based products such as soy milk, soy drinks, okara, tofu, and soy cheeses [22,29]. The second most abundant source of this isoflavone is represented by legumes with an average content ranging from 0.2 and 0.6 mg/100 g [21,30,31]. Fruits, nuts, and vegetables contain variable amounts of genistein, in the range of 0.03 to 0.2 mg/100 g [31]. A more comprehensive list of genistein content in food classes is available online [26].

Various treatments (such as hulling, flaking, and defatting) are used to isolate soya proteins from the soya bean [33]. Fermentation is a common low-cost process to improve the bioavailability of soy [34]. The isoflavone is extracted from soybean meal in organic solvents and genistin-containing fractions are converted to genistein by acid hydrolysis [35]. The products are solubilized in water and genistein is crystallized from the solution [36]. Recently, the methods for the isolation of genistein have been improved and have become more complex and sensitive [37]. Most of the separation and purification methods are based on thin-layer chromatography and other chromatographic techniques that are based on a solid stationary phase, such as semi-preparative and preparative HPLC [38,39]. In recent years, high-speed countercurrent chromatography (HSCCC) has become the method of choice for isoflavone preparation [38,40].

## 4. Bioavailability and Metabolism

While it is possible to produce synthetic forms of genistein to increase the supply [27,41], the main source for most people remains soy products in their diet [20,42].

In soybeans, soy foods, and natural sources, isoflavones are generally present as a mixture of glycoside conjugates linked to either glucose, malonyl, or acetyl derivatives of glucose in cell vacuoles [32,43,44,45,46,47]. In soybeans, the glycoside (sugar conjugate) form of genistein, known as genistin, is the more abundant while the biologically active and free form (aglycone) is present in small amounts [47,48]. Although studies on human bioavailability have produced conflicting results, at least partly due to the different formulations used, isoflavones generally exhibit low bioavailability [49,50]. However, compared to aglycones, the glycosylated forms of isoflavones tend to be more water-soluble and polar and, for this reason, they are poorly absorbed from the gastrointestinal tract and show weak biological activity [49,50]. Hydrolysis of the carbohydrate moiety is required to ensure the bioavailability of these forms [44]. Various reports, however, suggest that the oral bioavailability of genistin is higher than that of genistein, as the former compound is found more rapidly in plasma than the latter [45,51,52].

Free genistein concentrations in soy users’ blood are in the low nanomolar range [15]. Instead, isoflavone aglycones can be rapidly transported from the jejunum by non-ionic passive diffusion but they have a low bioavailability due to their lipophilicity [44]. Indeed, in humans, isoflavones appear in blood plasma faster and in higher concentrations after oral aglycone rather than glycoside administration [53]. The isoflavones are hydrolyzed by the gut microbiota [54] or gut wall enzymes (such as phloridzin-lactate hydrolase and other intestinal β-glucosidases [35,55]) and their sugar moiety is released [56,57]. Subsequently, genistein and its derivatives are metabolized in the gut or liver to glucorinidated and sulfated forms [56,57]. They are then transported back into the intestinal lumen and the blood [16,17,58,59]. The conjugation of genistein with sugars ensures rapid elimination by biliary and urinary excretion [17,60]. Several approaches are being used to optimize the solubility and bioavailability of genistein including nanostructured lipid carriers or other molecular carriers, complexation with chemically modified cyclodextrins, or co-crystal engineering [58,59,60,61].

## 5. Genistein and Angiogenesis

**Figure 2:**
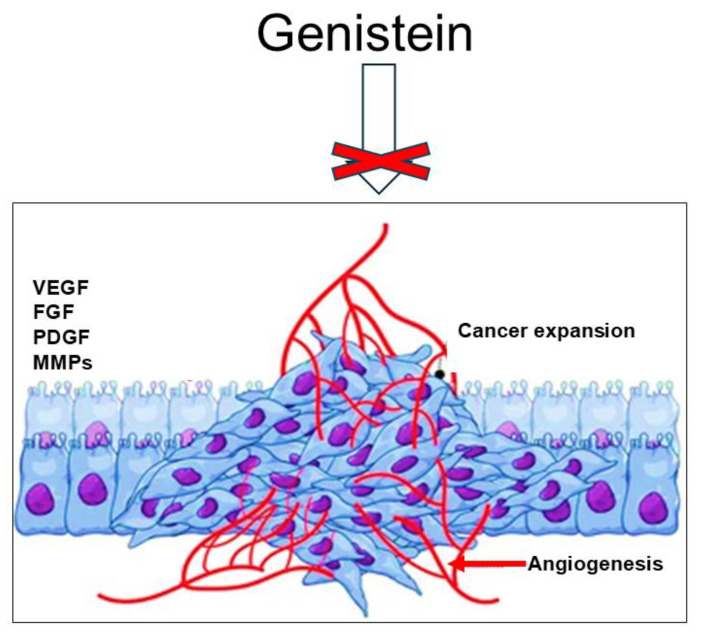
Schematic representation of the inhibitory effect of genistein on angiogenesis. The isoflavonoid suppresses tumor neovascularization by interfering with the action of specific growth factors (such as VEGF, FGF, and PDGF) and matrix-degrading enzymes, including MMPs. Full names of the proteins and other abbreviations can be found in the Abbreviations section.

Angiogenesis involves the dynamic formation of new blood vessels from a preformed vascular system [62]. Angiogenesis is tightly regulated by pro- and antiangiogenic mediators and primarily occurs during embryonic development. In healthy adults, it is generally infrequent and is associated with specific physiological events, such as the female menstrual cycle and pregnancy [62,63,64,65]. In the tumor context, the aberrant metabolism coupled with the typical hypoxic conditions leads to proangiogenic factor overexpression, including vascular endothelial growth factor (VEGF), platelet-derived growth factor (PDGF), fibroblast growth factor (FGF), and matrix metalloproteases (MMPs) [66,67]. Consequently, angiogenesis is a crucial process during all phases of the tumor disease promoting cancer cell proliferation, migration, and invasion, as well as metastases formation [68,69]. Therefore, targeting tumor angiogenesis appears to be a strategic method to interfere with cancer expansion [69]. As part of its chemopreventive properties, genistein functions as a potent antiangiogenic agent and has demonstrated efficacy in managing breast and prostate cancers [12,13,14,16]. For example, as extensively reviewed by Varinska et al. (2015), by interfering with the activity of various proangiogenic mediators including VEGF, epithelial growth factor receptor (EGFR), MMPs, nuclear factor-kappa B (NF-κB), phosphatidylinositol 3-kinase (PI3K)/protein kinase B (PI3K/Akt), and GEN (ERK1/2) signaling pathways, GEN strongly inhibited angiogenesis in human breast cancer (BC) [70]. Experimental evidence demonstrates the antiangiogenic properties of the flavonoid, showing that genistein can bind to hypoxia-inducible factor-1α (HIF-1α) in an in vitro model of human BC [71]. HIF-1α signaling is a crucial hub implicated in activating the angiogenic switch, sustaining tumor growth and metastases diffusion. Thus, it is reasonable that targeting the HIF-1α/HIF axis may represent a promising antiangiogenic and anticancer therapeutic approach [72]. Furthermore, in line with the action of the flavonoid explained above, by using a dedicated multiplex-array assay, Uifalean and co-workers (2018) showed genistein dose-dependently hindered C-X-C motif chemokine ligand 16 (CXCL16) and vascular endothelial growth factor-A (VEGFA) secretion in BC in vitro (in MCF-7 estrogen-dependent and MDA-MB-231 estrogen-independent cell lines) [73]. Interestingly, both CXCL16 and VEGFA not only trigger angiogenesis but also promote metastasis [73,74]. More investigations reveal that genistein inhibits angiogenesis in other tumor types [75,76]. For example, it was found that the compound repressed the expression/secretion of different angiogenic factors (including VEGF and PDGF) and matrix-degrading enzymes (such as urokinase-type plasminogen activator (uPA), MMP-2, and MMP-9) in human bladder cancer cells, upregulating angiogenesis inhibitor levels (such as plasminogen activator inhibitor-1 (PAI-1), angiostatin, endostatin, and thrombospondin-1 (TSP-1)) [75]. In addition, genistein treatment caused a relevant decrease in VEGF mRNA expression in thyroid carcinoma cells [76]. Of note, this suppressive effect was more emphasized in the case of the genistein–thymoquinone combination [76]. Taken together, this evidence emphasizes genistein’s inhibitory effect on tumor angiogenesis as part of its chemopreventive efficacy (Figure 2).

## 6. Genistein Inhibits Cancer Invasion and Metastases

Extracellular matrix (ECM) remodeling and degradation are critical in cancer progression [77,78]. Matrix-degrading enzymes, including Ca^2+^- and Zn^2+^-dependent endopeptidases, namely MMPs and the uPA system, are the main mediators responsible for ECM molecule lysis [79,80,81,82]. As part of its chemopreventive action, by modulating the different molecular signaling pathways discussed below, genistein negatively influences the expression/activation of matrix-lysis enzymes, thereby acting on multiple aspects of cancer metastasis [79,83]. In this regard, a study by Kousidou and colleagues demonstrated that genistein downregulates the mRNA expression of several members of the MMP family in human BC cells, including estrogen-receptor-negative MDA-MB-231 and MCF-7 cell lines. Functionally, this reduced invasion of the cancer cells [82]. In addition, genistein dose-dependently efficiently inhibits transforming growth factor beta1 (TGF-β1)-induced invasion and metastatic potential of human pancreatic cancer in vitro by downregulating MMP-2 and uPA expression [84]. The anti-invasive impact of genistein on pancreatic cancer cells was also supported by the targeting of Forkhead box protein M1 (FOXM1) as shown by Wang et al. (2010) [85]. Furthermore, Xiao and co-workers demonstrated that the isoflavone inhibited (in a dose-dependent manner) the invasiveness of human colon–rectal cancer (CRC) cells, as well as metastasis formation in murine orthotopic implantation models, through the selective suppression of the proangiogenic marker Fms-related tyrosine kinase 4 (FLT4) (i.e., VEGFR2) and MMP-2 [86]. Additionally, Hussain et al. (2021) revealed that genistein, in a time-dependent manner, inhibited the invasive ability of HeLa cells by modulating MMP-9 and tissue inhibitor of metalloproteinases 1 (TIMP-1) [87].

The invasiveness of cancer cells is closely related to the abundance of focal adhesions (FAs), large and dynamic protein complexes, that link the cytoskeleton to the ECM; of note, FAs particularly accumulate in specialized membrane protrusions (i.e., invadopodia and podosomes) that contribute to the lysis of ECM through the action of MMPs [88]. Focal adhesion kinase (FAK) and paxillin are the main FA-associated kinases, and their activation is crucial in controlling cell migration and invasion [89]. Given this, genistein, by downregulating p125FAK, inhibited the metastatic activity of hepatocellular carcinoma (HCC) cells (Bel 7402) coupled with angiogenesis suppression in a murine xenograft model [90].

As already emphasized, genistein is recognized as a phytoestrogen due to its ability to interact with ERs [91]. Not surprisingly, its biological functions are largely modulated by the ERα and ERβ subtypes [92]. On this premise, Chan et al. (2018) noticed that genistein (in a dose-dependent trend) significantly reduced both migration and invasive ability of ER-positive ovarian cancer cells (SKOV-3 positive for ERα and A2780CP expressing ERβ) by suppressing FAK signaling [93]. Similarly, the invasion ability of human choriocarcinoma cells (JAR cells) was silenced by genistein via ERβ binding, followed by metastasis-associated proteins 3 (MTA3)/Snail/E-cadherin pathway activation [94].

The mitogen-activated protein kinase (MAPK) pathway transmits extracellular signals from the membrane to intracellular compartments and it is involved in several cellular processes including cell proliferation, migration, invasion, as well as cell death [95]. A study by Chen and colleagues demonstrated that genistein, in a dose-dependent manner, inhibited the metastatic potential of human cervical cancer cells (HeLa) in vitro by interfering with MAPK signaling pathways and FAK-paxillin activation [96]. Furthermore, by inhibiting MMP-9 expression and interrupting specific transductors of the MAPK pathway (namely MEK/ERK and JNK signaling pathways), genistein (in a dose-dependent manner) was able to suppress both migration and invasion of squamous cell carcinoma in vitro (SK-MEL-28 cells) [97].

Interestingly, in 16F10 melanoma cells, different doses of genistein were found to affect cancer cell invasiveness. High doses of the phytoestrogen (100 μM), by inactivating the FAK/paxillin signaling cascade, inhibited the cell adhesion, migration, and invasion triggering apoptosis; on the contrary, a low dosage of genisetin (12.5–50 μM) significantly promoted both invasion and migration of melanoma cells by activating the FAK/paxillin and MAPK signaling pathways [98]. Moreover, using a B16 mouse model of murine melanoma (B164A5 melanoma cell line and C57BL/6J mice), genistein showed antimetastatic activity and reduced the tumor’s size [99].

Tetradecanoylphorbol-13-acetate (TPA) is one of the most widely used agents to study the mechanisms of carcinogenesis and metastasis; in fact, it induces MMP expression/activation by modulating different signaling pathways [100]. Wang et al. (2014) observed that genistein blocks TPA-mediated metastasis via the downregulation of MMP-9 and epidermal growth factor receptor (EGFR) followed by the suppression of nuclear factor-κB (NF-κB) and activating protein-1 (AP-1) transcription factors and inhibition of MAPK, IκB, and PI3K/Akt signaling pathways in an HCC model [101].

Recently, Khongsti and co-workers found that human BC cells (MDA-MB-435 and MDA-MB-231) treated with genistein showed a reduction in osteopontin (OPN) secretion associated with increased phosphorylation of ERK1/2 and mitogen-activated protein kinase kinase 1/2 (MEK1/2) and upregulation of a member of the NAD (+)-dependent histone deacetylase family, namely SIRT1. Thus, the authors speculated that OPN inhibition via genistein may be epigenetically regulated by MAPK-pathway-induced SIRT1 [102]. Notably, OPN plays a pivotal role in cancer [103].

In prostate cancer (PC), the effects of genistein are rather inconsistent [104,105,106,107]. As a matter of fact, in a study by Touny et al. (2009) genistein curiously promoted and exacerbated the metastatic propensity of undiagnosed early-stage human PC through an estrogen- and PI3K-dependent mechanism, also involving OPN upregulation [104]. Conversely, in another investigation, genistein inhibited PC invasion and MMP-2 activation, switching off TGF-β-mediated phosphorylation of MAPK-activated protein kinase 2 (MAPKAPK2) and heat shock protein 27 (HSP27), both downstream regulators of p38MAP kinase signaling [105]. The antimetastatic effect of genistein on PC cells (1532CPTX, 1532NPTX, 1542NPTX, 1542CPTX, PC3, and PC3-M cell lines) was further supported by Xu et al. (2009) who showed suppression of MMP-2 by targeting the mitogen-activated protein kinase kinase 4 (MEK4) [107]. In this circumstance, the authors also noted the clinical relevance of genistein treatment in inhibiting signaling pathways involved in PC invasion. By analyzing normal prostate epithelial cells isolated from the prostate tissue of patients enrolled in a prospective, randomized phase II trial, they observed lower levels of MMP-2 expression compared to cells from untreated patients [107]. To address the controversial impact of genistein treatment on PC, Nakamura’s group developed a clinically relevant xenograft model generated from a patient’s prostatectomy specimen and demonstrated a prometastatic effect of the flavonoid. Thus, the authors speculated that genistein may have heterogeneous antimetastatic effects correlated with differential ERβ expressions among patients [106]. Additionally, as discussed below, non-coding RNAs (ncRNAs), including different microRNAs (miRs, 18–25 nt), that act as oncogenes and/or tumor suppressors in different cancers, can be regulated by genistein [108]. Indeed, as discovered by Chiyomaru and co-workers, genistein, both in vitro and in mouse models, by upregulating miR-574-3p expression and affecting related signaling pathways, decreased the invasion and metastatic activity of androgen-independent PC cell lines (PC3 and DU145, both expressing the ERβ subtype) [109].

Extensive evidence suggests that a dysregulated expression of KCNK9 (a member of the two-pore domain potassium (K2P) channel family) supports cancer progression [110,111]. Pertinently, a study by Cheng’s group demonstrated that genistein, by downregulating KCNK9 expression, acted on the Wnt/β-catenin signaling pathway, suppressing the malignant phenotype of human colon cancer [112].

The catalytic subunit of DNA-PK kinase (DNA-PKcs), which plays a major role in DNA damage signaling repair, also shows a proinvasive action [113]. As radiotherapy increases the invasive tendency of DNA-PKcs-positive glioblastoma multiforme (GBM), it has recently been shown that genistein can specifically bind to DNA-PKcs, suppressing the DNA-PKcs/Akt2/Rac1 signaling pathway, thereby successfully inhibiting the radiation-induced invasiveness of GBM cells in vitro and in vivo [114].

Epigenetic plasticity (i.e., DNA methylation/demethylation, histone modifications, as well as short and long ncRNA involvement) can contribute to cancer onset and progression [115]. For example, the methylation in the promoter of the gene coding Wnt inhibitory factor 1 (WIFI) favors cancer development by switching off the Wnt/β-catenin signaling pathway [116]. Pertinently, Zhu and co-workers found that genistein inhibited the invasion and migration of colon cancer cells (HT29) by inducing WIF1 gene demethylation, thereby restoring its activity. These effects were also supported by the regulation of cancer cell invasion-associated genes, including MMP-9, MMP-2, TIMP-1, E-cadherin, and Wnt signaling pathway mediators including β-catenin, c-Myc proto-oncogene protein, and cyclin D [117]. As mentioned above, the epigenetic modulator miRs can represent a target of genistein [108]. Consistently, it was observed that the phytochemical, by targeting miR-27a, inhibited invasion, suppressed cell growth, and induced apoptosis in pancreatic cancer cells [118]. In addition, Hirata et al. (2021) demonstrated that isoflavones, by regulating miR-1260b expression, inhibited Wnt signaling effector genes such as secreted frizzled-related protein 1 (sFRP1), Dickkopf 2 (Dkk2), and Smad4 in renal cancer cells (786-O, A-498, Caki-2 cell lines); functionally, cancer cell invasiveness together with proliferation and apoptosis phenomena was significantly decreased [119]. Another interesting study showed that genistein interrupted the metastatic activity of human colorectal cancer (CRC) by suppressing the interplay between the long ncRNA TATTY18 and the Akt pathway [120]. In more detail, genistein-treated CRC cells (SW480), among other phenomena, showed downregulated expressions of TATTY18 and reduced cell migration, coupled to a decrease in glucocorticoid-regulated kinase 1 (SGK1) and reduced Akt and p38-MAPK phosphorylation; these results were confirmed in vivo using genistein-treated tumor-bearing nude mice [118]. Circular RNAs (circRNAs), like other ncRNAs (i.e., micro- and long-ncRNAs), can act as oncogenes [121,122,123]. Recently, members of the flavonoid family have been reported to interfere with cancer progression by targeting specific circRNAs [122,123]. FOXM1, a cell-cycle-regulating transcription factor upregulated in cancer, has the role of maintaining malignant hallmarks by modulating the expression of target genes at the transcriptional level [124]. Interestingly, Yu et al. (2021) discovered that genistein exposure regulated non-small cell lung cancer (NSCLC) cell migration and invasion by decreasing the circRNA circ_0031250; in parallel, they showed that miR-873-5p is a target of circ_0031250 to finally conclude that genistein restricts NSCLC invasiveness and progression by involving the circ_0031250/miR-873-5p/FOXM1 axis [125].

It has been observed that genistein can synergistically hinder cancer spreading when combined with other compounds including specific phytochemicals. For example, a mixture of genistein and retinoic acid (ATRA) significantly inhibits the invasion ability of human adenocarcinoma cells (A549 cell line) by downregulating the transmembrane protein mucin 1 (MUC1) as well as intercellular adhesion molecules-1 (ICAM1) expression [126]. Furthermore, adding rottlerin to genistein on neuroblastoma cells (SH-SY5Y and Kelly) led to notable decreased levels of eukaryotic elongation factor 2 kinase (EF2K) which is known to influence invasion/metastasis and the integrin/Src/FAK axis [127].

**Figure 3:**
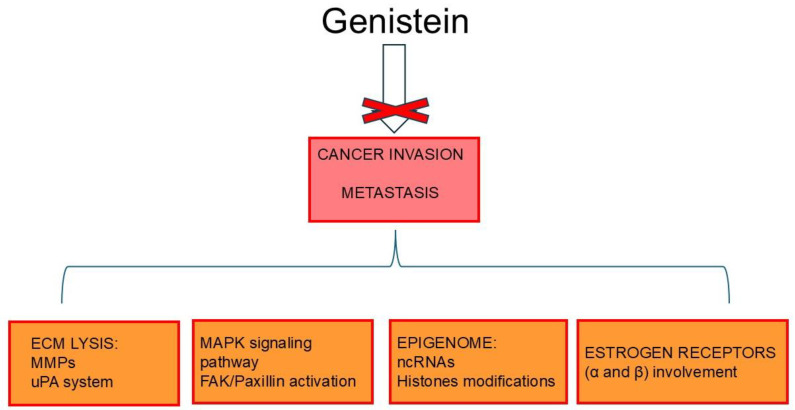
Influence of genistein on cancer progression. Genistein abrogates cancer invasion and metastasis by interfering with multiple mechanisms including EMC lysis, MAPK/FAK/paxillin activation, epigenetic changes (ncRNA engagement and histone modifications), and estrogenic receptor involvement. Full names of the proteins and other abbreviations can be found in the Abbreviations section.

To sum up, genistein can interfere with cancer invasion and metastasis by acting on multiple molecular mechanisms (Figure 3).

## 7. Genistein and Epithelial Mesenchymal Transition

EMT is a dynamic and complex process by which epithelial cells lose their junctions and polarity and acquire a migratory mesenchymal phenotype, involving reorganization of the cytoskeletal scaffold [128,129]. Thus, typical epithelial markers including E-cadherin (CDH1), claudins, zonula occludens-1 (ZO-1), desmoplakin, and plakophilin are replaced by mesenchymal proteins such as N-cadherin (CDH2), vimentin (Vim), and α-smooth muscle actin (α-SMA) [128]. Multiple and interacting signaling pathways orchestrate the EMT phenomenon, such as tumor growth factor-β (TGF-β), epidermal growth factor (EGF), FGF, PDGF, Notch, Wnt/β-catenin, PI3K-Akt, FAK/paxillin, MAPK signaling, and the Hippo-Yes-associated protein (YAP)/PDZ-binding motif (TAZ) pathways, as well as several ncRNAs [128,130,131,132]. In synergy with the above molecular signals, numerous transcription factors such as Snail, Slug, Twist, NF-κB, HIF1/2 and zinc finger E-box binding homeobox 1/2 (ZEB1/2), and basic helix–loop–helix (bHLH), are involved in EMT [133]. Physiologically, EMT plays a crucial role in organogenesis and tissue repair [134]. In the tumor context, this process contributes to cancer malignancy by eliciting cell invasion, cancer stem cell (CSC) grouping, and drug resistance and supporting metastasis formation [135]. Therefore, inhibiting or reversing EMT may be a clinically relevant strategy to prevent cancer spread. As explored by Hsieh et al. (2020), genistein was able to suppress (in a dose-dependent trend) the prometastatic propensity of human head and neck cancer (HNC) coupled with a decrease in multidrug resistance by regulation of typical EMT markers (i.e., E-cadherin, Vim, Slug, and ZEB1) [136]. Additionally, by introducing an in vivo model, the authors concluded that genistein inhibited the aggressiveness of the HNC cells by perturbing the miR-34a/RTCB axis [136]. Another study showed that genistein, in combination with a specific miR-223 inhibitor, reversed the EMT process in gemcitabine-resistant pancreatic cancer cells, as supported by a downregulation in the expressions of mesenchymal markers (such as Slug, Vim, Snail, ZEB1, and ZEB2), thus enhancing the drug sensitivity and inhibiting cell motility and invasion [137]. As previously highlighted, genistein as a phytoestrogen can exert multiple biological functions by binding to ERs, including chemoprevention [16,18,19,21]. E2, as well as the typical endocrine-disrupting chemicals (EDCs) such as bisphenol A (BPA) and nonylphenol (NP), has been shown to promote EMT and invasiveness in estrogen-responsive cancers, including ovarian cancer [138]. On these premises, Kim and co-workers found that genistein was effective in reversing the ER-mediated EMT process activated by E2, as well as BPA and NP, in human ovarian cancer cells (BG-1) and reducing the resulting increase in expression of invasion markers such as MMP-2 and cathepsin D. In a parallel setting, the same investigators showed that the phytoestrogen was also able to reactivate the TGF-β signaling pathway in the BG-1 cells after its suppression by the compounds mentioned above [138]. Furthermore, in human HCC, genistein dose-dependently reversed EMT in vitro by partly suppressing the nuclear factor of activated T cell 1 (NFAT1) expression and thus showed antimetastatic activity in nude mice bearing liver orthotopic tumor implants [139]. In human colon cancer cells (HT-29) genistein (200 µgmol/L) decreased the expression of typical EMT molecules (i.e., N-cadherin, Snail2/Slug, ZEB1, ZEB2, FOXC1, FOXC2, and TWIST1), perturbed the Notch-1/NF-κB axis, and induced apoptosis [140]. Moreover, in human pancreatic cancer cell line Panc-1, genistein, through a Smad4-dependent signaling pathway, in a dose-dependent fashion, suppressed TGF-β1-induced EMT and invasiveness [84]. As discussed in other contexts of this review, the role of the genistein in PC remains disputable [104,105,106,108,109]. However, using dedicated PC cell lines (highly metastatic IA8-ARCaP cells and LNCaP/HIF-1α cells that stably overexpress HIF-1α), Zhang et al. (2008) found that low-dose genistein (0.2–15 μmol/L) inhibited invasion in vitro by reversing the EMT process [141]. Differently, a study performed by Du and co-workers found that genistein treatment inhibited endothelial growth factor (EGF)-dependent EMT in laryngeal cancer cells (Hep-2 cell line), inhibited cancer cell growth and migration, and promoted apoptosis; moreover, all these antitumor effects were more evident when trichostatin A (TSA) was added to genistein [109]. Another investigation demonstrated that genistein treatment of papillary thyroid carcinoma (PTC) cells, by preventing nucleus translocation of β-catenin, was able to block the EMT process as documented by alteration of EMT-related modulators (i.e., E- and N-cadherin; Vim and Snail), suppressed cell cycle/proliferation, and promoted cell death [142].

**Figure 4:**
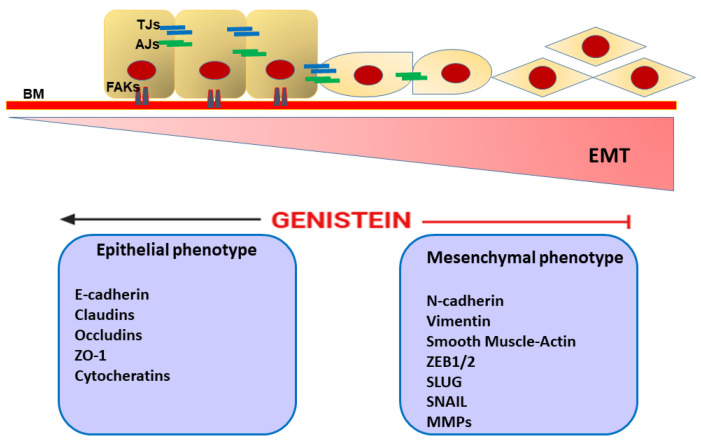
Picture showing the main molecular targets genistein interferes with during cancer-related EMT. Specific signaling pathways and transcription factors are mentioned in the epithelial phenotype box and mesenchymal phenotype box, respectively. Of note, genistein drives cytoskeleton rearrangement coupled to cellular junction disintegration. TJs: Tight junctions; AJs: Adherent junctions; FAKs: Focal adhesions (including integrins and paxilin). Full names of the proteins and other abbreviations can be found in the Abbreviations section.

Taken together, this experimental evidence suggests that genistein, by acting on multiple EMT-related mechanisms, can effectively contribute to blocking cell invasion and the metastatic propensity of different tumor entities (Figure 4).

## 8. Genistein Eradicates Cancer Stem Cells

CSCs constitute a cluster of highly invasive cancer cells able to trigger tumorigenesis, supporting the metastatic cascade [143]. Like normal stem cells, CSCs are capable of self-renewal and differentiation [144]. However, various aberrant signaling pathways are involved in the maintenance and propagation of this tumor subpopulation, including TGF-β, Wnt/β-catenin, Notch, Hedgehog, PI3K/Akt/mTOR, NF-κB signaling, as well as the Hippo-YAP/TAZ pathways [145,146,147,148]. Furthermore, several biomarkers, including cell surface molecules (i.e., cluster of differentiation (CD) 24, CD90, and CD133), and various transcription factors, such as octamer-binding transcription factors 3 and 4 (Oct-3 and -4), Nanog, and SEX-determining region (SRY) homology box 2 (Sox2), are frequently used to identify and isolate CSCs [149]. A peculiar aptitude of CSCs shown in in vitro conditions is their ability to form “spheroids” (known as mammospheres in the case of breast CSCs); moreover, CSCs have an instrumental role in conferring drug resistance as well as tumor recurrence; thus, given their potent role in tumor aggressiveness, CSCs represent a considerable target for cancer therapy [150].

**Figure 5:**
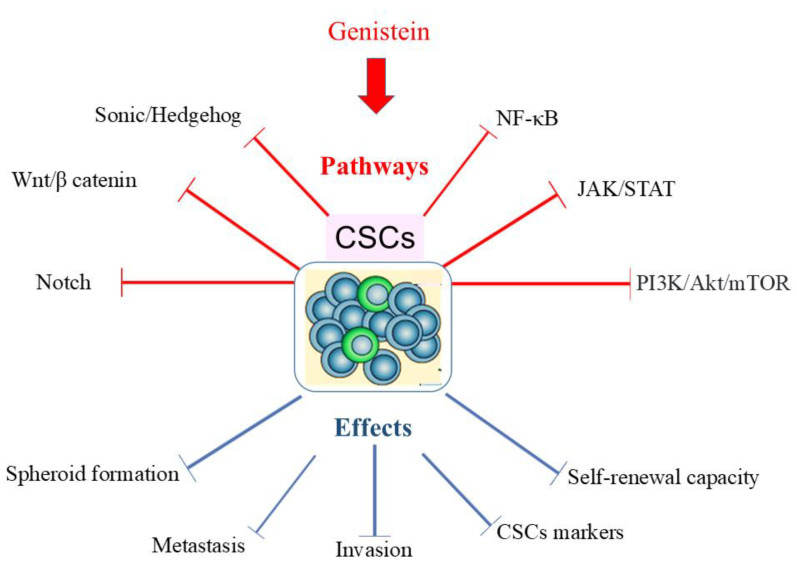
Diagram showing the main repercussions of genistein in CSC behavior. Genistein treatment affects several signaling pathways (i.e., Sonic/Hedgehog, Wnt/β-catenin, Notch, NF-κB JAK-STAT, PI3K/Akt/mTOR signaling) that are involved in the maintenance of self-renewal capacity, stemness markers, invasion and metastasis propensity, as well as in the aptitude to form spheroids. Full names of the proteins and other abbreviations can be found in the Abbreviations section.

Recent studies have highlighted the potential of various phytochemicals, such as isoflavones, to interfere with numerous signaling pathways involved in CSC propagation, effectively reducing their ability to metastasize [151]. In this scenario, it has been demonstrated that genistein, by acting on specific signaling pathways (i.e., Sonic Hedgehog (SHH), Wnt/β-catenin, Notch, NF-κB JAK-STAT, PI3K/Akt/mTOR signaling), affects CSC proliferation, thereby inhibiting tumor invasiveness and metastasis (Figure 5) [152]. Particularly, it is postulated that genistein exerts its anticancer effects by modulating specific signaling pathways such as the Wnt/β-catenin, Notch, and Hedgehog pathways, which are crucial for stemness maintenance and self-renewal [108]. Moreover, genistein is associated with several pharmacological activities, including inhibition of various kinases, transmembrane channels, and other crucial molecular mechanisms [153]. For instance, topoisomerase II, tyrosine kinases, MAPK, ATP-binding cassette (Abc) transporters, P13K/Akt, polo-like kinase 1 (PLK1), SHH, and Wnt/β catenin signaling pathways are all mechanisms impaired by the above-mentioned isoflavonoid [21,153,154].

Specifically, these activities, together with induction of cell cycle arrest and apoptosis, prevention of reactive oxygen species (ROS), resolution of inflammation, inhibition of angiogenesis, EMT, regulation of steroid hormones, and specific metabolic pathways, contribute to the anticancer properties of genistein [153,155,156,157].

Originally, among other effects, genistein was recognized as a key compound with wide-ranging medicinal activities and has also been used to limit cancer invasiveness [152]. Specifically, genistein was found to reduce PC stem cells both in vitro and in xenograft mice by inhibiting the Hedgehog pathway and interfering with CD44 expression; ectopically, tumor growth was found to be effectively reduced [152]. As clarified above, CSCs can form spheroids in vitro, preserving, among other stem-like properties, immortality and invasiveness. Thus, in the above experimental context, genistein dose-dependently significantly inhibited tumorsphere formation (22RV1- and DU145-derived stem cell formation) [152]. Furthermore, in a study performed by Fan and co-workers, it was demonstrated that genistein exerts multiple effects on MCF-7 BC cells, not only suppressing proliferation and inducing apoptosis but also specifically inhibiting the CSC subpopulations with consequent inhibition of mammosphere formation through downregulation of the Hedgehog–Gli1 pathway [158]. Moreover, the phytoestrogen induced BC stem/progenitor cell differentiation by interacting with ER-expressing cancer cells through a paracrine mechanism correlated with PI3K/Akt and MEK/ERK signaling [159]. Yu et al. (2014) found that genistein markedly reduced the Gli1 level, which is considered a mediator of the SHH signaling pathway; such a mechanism involves specific CSC hallmarks of gastric cancer (GC) including CD44 expression, stem-cell-related genes (i.e., Oct-4, Bmi, Nestin, and adenosine triphosphate (ATP)-binding cassette efflux transporter G2(ABCG2)), spheroid formation, as well as migration and invasion propulsion [160]. Of note, as observed by Huang’s group, genistein treatment also alters the resistance of CSCs to chemotherapeutic drugs such as 5-fluorouracil (5-FU) and cisplatin, and it can also reduce tumor size in animal models of GC cells [161]. Similarly, more recent studies demonstrate that genistein inhibits proliferation and induces apoptosis by inhibiting the SHH signaling pathway in CSCs derived from human renal cancer [162]. The same SHH-targeting mechanism was found in human nasopharyngeal cancer stemness eradication, including the arrangement of tumor spheroids [163]. In addition, many researchers agree that 7-difluoromethoxy-5′,4′-di-n-octyl genistein, a genistein analog characterized by high bioavailability, can effectively eradicate CSCs derived from GC and ovarian cancer by multiple mechanisms, including inactivation of Akt, ERK, and NF-κB signaling, downregulation of FOXM1, as well as the propensity of stem-like cells to originate spheroids and suppress EMT [164,165,166]. Fu et al. (2020) showed that genistein (20 and 40 µM) inhibited the sphere formation aptitude of CSCs derived from human NSLC cells, which was associated with a decreased expression of stem-cell-related markers such as CD133, CD44, Bmi1, and Nanog. In a parallel setting, the authors found that genistein also suppressed the migratory and invasive activities of these specific CSCs by modulating manganese superoxide dismutase (MnSOD) and FoxM1 signaling pathways [167]. Furthermore, inhibition of PI3K/Akt signaling, coupled with overexpression of phosphatase and tensin homolog deleted on chromosome 10 (PTEN), has been shown to be a relevant pathway by which CSC behavior can be controlled by certain dietary factors, including genistein [168]. Similarly, previous studies by Montales et al. (2012) demonstrated that genistein inhibits the formation of CSC spheres originating from human breast tumors, showing that the lowest dose of genistein (40 nM) consistently attenuated the formation of primary and secondary mammospheres from transformed cell lines and primary epithelial cells isolated from BC cells (MCF-7 (expressing ERα) and MDA-MB-231). In contrast, a supraphysiological dose of genistein (2 μM) was less effective in eliciting a similar biological outcome [168]. Additionally, for a specific MDA-MB-231 subpopulation expressing CD44 and epithelial-specific antigen (ESA) (being particularly enriched in CSC content), both 2 μM and 40 nM doses of genistein were effective in suppressing mammosphere formation. Furthermore, the inhibition of PI3K/Akt signaling, coupled with PTEN overexpression, has been shown to be a relevant pathway by which CSC behavior can be controlled by dietary factors, including genistein [168].

Given the potent anticancer effects of genistein, in particular targeting the CSC phenotype, recent clinical studies have been designed to investigate the preventive and therapeutic efficacy of this natural compound in different human tumors, including CRC (ClinicalTrials.gov NCT01985763 nct.gov, 2019), PC (ClinicalTrials.gov NCT01126879 nct.gov, 2019), BC (ClinicalTrials.gov NCT00290756 nct.gov, 2017), and urothelial cancer (ClinicalTrials.gov, NCT00118040 nct.gov, 2017, NCT01489813 nct.gov, 2018).

Thus, based on the above experimental evidence, it is reasonable to ponder genistein as a useful drug to attenuate the aggressiveness of CSCs.

## 9. Genistein and Cell Cycle Arrest

The cell cycle is the ordered and finely regulated sequence of events that occur in cells that are about to divide [169,170]. Progression through each step of the cell cycle is controlled by checkpoint proteins belonging to the cyclin-dependent kinase (CDK) family [170,171]. Disorders that disrupt the CDK family of proteins deeply affect the rate of cell division and are often implicated in the development of primary tumors [172]. DNA damage causes a pause in the cell cycle to allow for repair mechanisms before the cell is committed to the subsequent steps [169,170]. Notably, DNA damage checkpoints can be divided into those controlled by the tumor suppressor and transcription factor p53 and those ultimately under the control of the checkpoint kinase 1 (Chk1) [170]. Several proteins are involved in cell cycle checkpoint activation pathways induced by DNA damage and DNA double-strand breaks, including mediator of DNA damage checkpoint protein 1 (MDC1), ataxia telangiectasia mutated (ATM), ATM- and Rad3-related (ATR), Chk1, checkpoint kinase 2 (Chk2), and PLK1 [173,174,175]. Genistein has been shown to modulate several cellular pathways and one of the most studied is the signaling cascade that controls cell cycle arrest [154,176,177,178].

Concentrations of genistein between 5 and 200 µM have been shown to induce cell cycle arrest in different cancer cell lines although the mechanisms are not fully understood [142,179,180,181,182,183,184,185] as these concentrations, even if theoretically achievable, are much higher than those found in the bloodstream after food intake [15].

The extensive research on BC and PC evidenced the cell cycle arrest induced by genistein during the G2/M, G0/G1, and G1/S phases [186,187,188,189,190]. The analysis of differentially expressed genes performed on MCF-7 BC cells after treatment with genistein revealed a strong dose-dependent alteration in the expression of genes involved in cell cycle control, such as glioma pathogenesis-related protein 1 (GLIPR1), cell-division cycle protein 20 homolog (Cdc20), budding uninhibited by benzimidazole 1 (BUB1), mini-chromosome maintenance (MCM) complex 2, and cyclin B1 (CCNB1) [191]. Fang et al. (2016) showed that in the human triple-negative BC (TNBC) cell line MDA-MB-231, genistein affects various molecular processes during cell cycle progression, including DNA replication, cohesion complex cleavage, and kinetochore formation, through regulation via phosphorylation at 332 different sites on 226 proteins [192]. Consistently, in MDA-MB-435S, MDA-MB-468, and MCF-7 BC cells genistein induced a concentration-dependent accumulation of cells in the G2/M phase [193,194,195]. The same results were confirmed in MCF-7 and MDA-MB-231 BC cells where the DNA damage checkpoint (pATM) was activated and the levels of inactive pCdc25c and pCdc2 were upregulated, arresting cells in the G2/M phase [196]. In human BC MDA-MB-231 and SKBR3 cells, genistein exerted the same G2/M phase arrest in a dose-dependent manner and the molecular mechanism involved the inhibition of S-phase kinase-associated protein 2 (Skp2) and promotion of its downstream targets p21 and p27 [197]. G2/M phase arrest was confirmed in MDA-MB-231 cells after genistein administration in association with the downregulation of cyclin B1, Bcl-2, and Bcl-xL expression, possibly mediated by NF-κB activation via the Notch-1 signaling pathway [198]. Furthermore, genistein treatment of MDA-MB-231 BC cells resulted in G2/M cell cycle arrest as evidenced by a strong concentration-dependent reduction in the protein levels of cyclin B1, Cdk1, and Cdc25C; in particular, these results were mediated by a genistein-induced stable activation of ERK1/2 in a concentration- and time-dependent manner [199]. Similarly, genistein has been shown to induce G2/M phase block in BRCA1-impaired human BC MDA-MB-231 and HCC1937 cells by downregulating cyclin B1 levels due to genistein-induced suppression of seven-transmembrane receptor G protein-coupled receptor 30 (GPR30) activation and Akt phosphorylation/activation [200]. A similar mechanism involving the PI3K/Akt pathway was found in the PC cell lines PC3 and LNCaP, where 5 or 10 µM genistein induced a significant G2/M cell cycle arrest [201]. Co-treatment with selenite and genistein showed synergistic effects on G2/M cell cycle arrest, although the effect in PC3 cells was less than in LNCaP cells [201]. Higher levels of p21waf1 and Bax were detected in genistein-treated PC3 and LNCaP cells, while AKT phosphorylation was decreased only in PC3 cells with no change in total AKT protein levels [201]. Similarly, in PC cells (PC3 cell line), cell cycle analysis revealed a G2/M phase arrest induced by genistein treatment associated with a 1.1-fold increase in nuclear p21WAF1/Cip1 protein expression and a 14% decrease in nuclear cyclin B1 expression [202]. The combination of genistein and radiation had an even stronger effect on these cells than either treatment alone [202]. Furthermore, the increase in p21 detected in LNCaP and PC3 cell lines after genistein administration is associated with delayed mitosis and has been linked to transcriptional inhibition of PLK-1 [203]. In another human cell line, PCDU145, genistein blocked the cell cycle in the G2/M phase and induced the growth arrest and DNA amage-inducible 45 (GADD45) gene thorough its promoter [189]. The Gadd45 protein is a target gene of p53 and has been reported to play a role in cell cycle regulation [204,205]. Similarly, cell cycle arrest in the G2 phase in response to genistein treatment has been described in BC MCF-7 and PC cell lines (PC3), where genistein downregulated the mouse double minute 2 (MDM2) oncoprotein at the transcriptional level by interacting with the MDM2 promoter and at the post-transcriptional level by inducing MDM2 ubiquitination [188]. The inhibition of MDM2 expression by genistein was confirmed in PC3 xenografts [188].

Indeed, by flow cytometry analysis, genistein was confirmed to induce an accumulation of cells in the G2/M phase of the cell cycle in the PC cell lines LNCaP, DU145, and PC3 [206,207]. Gene expression analysis performed in genistein-treated cells showed that in DU145 cells, the cyclin-dependent kinase 4 (CDK4), cyclin-dependent kinase inhibitor 2A (CDKN2A), and minichromosome maintenance 4 (MCM4) genes were downregulated while only the SERTA domain-containing 1 (SERTAD1) gene was significantly upregulated [206]. In PC3 cells, genistein administration caused a significant decrease in minichromosome maintenance 3 (MCM3) mRNA expression and an increase in cyclin H (CCNH) [206]. In LNCaP cells, the baculoviral IAP repeat-containing 5 (BIRC5), cyclin B2, Chk2, CDC28, protein kinase regulatory subunit 1B (CKS1B), GTSE1, hairy-related 5 (HERC5), minichromosome maintenance 2 (MCM2), MCM4, and proliferating cell nuclear antigen (PCNA) genes were downregulated while, in CDK7, cyclin-dependent kinase inhibitor 1A (CDKN1A) and cyclin-dependent kinase inhibitor 1B (CDKN2B)p were upregulated after genistein treatment [206]. In line with this, a significant G2/M phase arrest was described in the genistein-treated DuPro androgen-insensitive PC cell line; these cells showed suppression of cyclins with concomitant induction of the tumor suppressor genes p21 (WAF1/CIP1/KIP1) and p16 (INK4a) [190]. In particular, genistein has been shown to increase active acetylated histones at p21 and p16 transcription start sites [190]. Two different studies showed that genistein-induced G2/M phase cell cycle arrest in T47D BC cells is mediated by the formation of 5,7,3′,4′-tetrahydroxyisoflavone (THIF), a product of genistein cellular metabolism [121,122]. THIF has been shown to cause cell cycle arrest through activation of ATR, which is a consequence of intracellular oxidative stress, GSH depletion, and increased DNA damage [119]. In addition, THIF induced inhibition of cdc2, phosphorylation of p53 and Chk1, and deactivation of cdc25C phosphatase [121]. Moreover, another study by Nguyen et al. (2018) attributed the effect of THIF to the phosphorylation of p38 MAP kinase, resulting in the inhibition of cyclin B1 and cdc2 activation [208]. Additionally, genistein and a hydantoin-derived antiandrogen–genistein conjugate caused a significant accumulation in the S-phase of LNCaP cells [209].

Although the effect of genistein has often been described in terms of arresting the G2/S phase of the cell cycle, some studies have shown a cell cycle arrest induced by genistein administration in BC cell lines in a different phase. In more detail, genistein induced a G0/G1 phase arrest in MCF-7 and MDA-MB-231 cell lines [210,211]. Lin et al. (2009) confirmed the ability of genistein to inhibit the entry of the BC cell lines HS578T, MDA-MB-231, and MCF-7 into the G0/G1 phase of the cell cycle [212]. Moreover, in the T47D cell line genistein administration resulted in G0/G1 phase cell cycle arrest only when cells reached confluence [213]. In PC LNCaP cells, genistein induced a G0/G1 cell cycle arrest through the increased expression of histone acetyltransferases responsible for the increased transcription of p21 and p16 suppressor genes which induce cell cycle arrest [190].

In MDA-MB-231 and MCF-7 cells, genistein induced the accumulation of cells in the G0/G1 phase, and co-treatment of cells with GEN and a potent steroid hormone precursor called 1,25-dihydroxycholecalciferol (1,25(OH)2D3) confirmed this result with a stronger effect on the MDA-MB-231 cell line [187]. Similarly, in the ER+/HER2-overexpressing BT-474 human BC cell line, genistein and tamoxifen monotherapy significantly increased the G1 phase cell population, and this G1 arrest effect was enhanced when the two molecules were used in combination [214].

**Figure 6:**
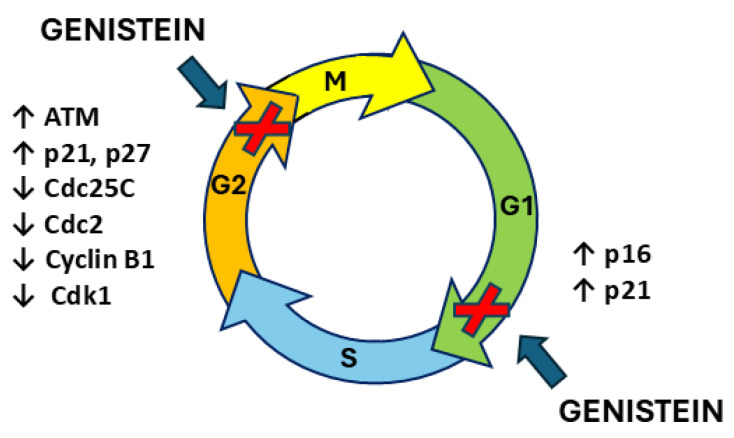
Genistein arrests the cell cycle of several human cancer cell lines in the G2/M or G1/S phase through the modulation of cell-cycle-related proteins. The main targets of genistein are indicated. Downward arrow ↓, decrease; Upward arrow ↑, increase. Full names of the proteins and other abbreviations can be found in the Abbreviations section.

In conclusion, genistein appears to have a wide range of effects on the cell cycle, which vary depending on the cell line and the phase of the cell cycle at the time of treatment [185,215]. As a potent modulator of cell-cycle-related proteins and a regulator of several molecular targets involved in cell cycle progression, genistein may have anticancer properties by modulating the effects of deregulated cell cycle checkpoints in cancer cells [185] (Figure 6).

## 10. Programmed Cell Death

Apoptosis is a programmed cell death (PCD) mediated by multiple signaling pathways triggered by cellular stress, DNA damage, immune surveillance, and other stressing cellular factors [216,217]. Two distinct signaling pathways—the extrinsic and intrinsic apoptotic pathways—trigger apoptosis in most tumor cells [217,218]. The extrinsic pathway involves the activation of death receptors such as Fas and tumor necrosis factor receptors (TNFRs), leading to the activation of caspase-8 and caspase-3 to induce apoptosis [218,219]. The intrinsic pathway is associated with changes in mitochondrial permeability, leading to the mitochondrial release of proapoptotic proteins such as B-cell lymphoma 2 (Bcl-2)-associated X protein (Bax), cytochrome c (cyt c), and apoptosis-inducing factor (AIF) into the cytoplasm and activation of caspase-9 and caspase-3, ultimately triggering apoptosis [218,219,220]. Caspase-3 is responsible for cleaving poly (ADP-ribose) polymerase (PARP) during cell death and is derived from both extrinsic and intrinsic pathways. Caspase-8 can cleave BH3-interacting domain death agonist (BID), a death-inducing member of the B-cell lymphoma 2 (Bcl-2) family, allowing crosstalk between the extrinsic and intrinsic apoptotic pathways [220]. The cleaved BID translocates to mitochondria and induces the release of cyt c, leading to caspase-9-dependent activation [218,220,221]. The ERK and NF-κB pathways can inhibit this apoptotic signal [218,222].

Cancer cells tend to evade cell death by activating antiapoptotic mechanisms, so cancer cell death can be achieved by reversing antiapoptotic processes [223,224,225]. Genistein has anticarcinogenic effects; in fact, several studies have shown that this isoflavone inhibits cell proliferation and induces death in several human cancer cells [18,108,176,183,226]. In this section, we will examine in detail the effects of genistein on the activation of apoptotic death in hormone-dependent cancers, such as prostate and breast cancers, which have been extensively studied [14,16,211,215,227].

### 10.1. Breast Cancer

The effects of genistein have been studied in BC MCF-7 cells through oligo microarray technology and the expression of multiple Bcl-2 family genes, which resulted in both pro- and antiapoptotic altered genes [228].

A study by Tophkhane et al. (2007) has shown that genistein-induced enhanced cell death and growth inhibition in MCF-7 cells overexpressing high levels of Bcl-2. Genistein administration resulted in increased levels of p85, a major subunit of cleaved PARP, membrane permeability changes, and cyt c release, suggesting that the enhanced activation of the caspase cascade involved in Bcl-2 overexpression mediates the sensitization of MCF-7 cells to genistein. Furthermore, the enhanced activation of the apoptotic process in Bcl-2-overexpressing cells is due to the isoflavone-induced accumulation of Bcl-2 and alteration of Bax anchoring in mitochondria [229]. Similarly, in MCF-7 cells, genistein-induced apoptosis led to a reduction in Bcl-2 expression and induction of Bax. In MCF-7 cells, both the expression of ERα and the proliferation were reduced after the administration of genistein [230]. Furthermore, high concentrations of genistein inhibited both normal MCF-10A and cancerous MDA-MB-231 ERα-negative breast cells, associated with p53- and p21-dependent apoptosis activation. Genistein increased proapoptotic proteins (phospho-p53 (p-p53) and p21) and decreased antiapoptotic proteins (Bcl-xL or cyclin B1) in breast cells [231]. These data were confirmed by the study of Ye et al. (2018) which showed that genistein significantly inhibited cell proliferation and induced pronounced apoptosis in the human BC cell lines MDA-MB-231 and SKBR3. The molecular mechanism involved downregulation and inhibition of S-phase kinase-associated protein 2 (Skp2) and upregulation of its downstream targets p21 and p27 [197]. Genistein promoted a p53-independent decrease in mouse double minute 2 (MDM2), which led to an increase in the half-life of p21 since this protein is a direct target of MDM2 for proteasomal degradation. Given that p21 is involved in genistein-induced apoptosis and G2 arrest in MCF-7 tumor cells, and that p21 protein is stabilized by genistein, it may mediate the antitumor effects of genistein [188]. Moreover, genistein-treated MCF-7, MCF-7-caspase-3 (MCF-7-C3), and T47D BC cells expressed low levels of the cancerous inhibitor of protein phosphatase 2A (CIP2A) which correlated with growth inhibition and apoptosis induction, as evidenced by PARP cleavage and the expression of functional caspase-3 in MCF-7-C3 and T47D [232].

Genistein has been shown to induce apoptosis via an ERα-independent pathway without the involvement of MAP kinase and Akt but associated with reduced Bcl-2/Bax ratio in MCF-7 cells [233]. Typically, genistein has been shown to inhibit the growth of MCF-7 cells and promote the apoptotic pathway through the inactivation of PI3K/Akt signaling, as evidenced by the decrease in phospho-Akt in treated cells, resulting in reduced expression of the downstream target HOX Antisense Intergenic RNA (HOTAIR) [234], a long ncRNA (lncRNA) implicated in cancer invasion and metastasis [235]. Furthermore, treatment with genistein resulted in a significant decrease in PI3K and AKT protein and a significant increase in Fas ligand, FAS-associated protein with death domain (FADD), cyt c truncated Bid, caspase-9, and caspase-3 in MCF-7 cells [236,237]. Similarly, genistein has been shown to induce the extrinsic FAS-receptor-dependent apoptosis pathway in MCF-7 cells engineered to overexpress oncogenic HER2 (MCF-7 HER2) and control vector cells (MCF-7 vec). Specifically, the phytoestrogen administration caused upregulation of p53 and levels of FAS receptor and cleaved caspase-8 and induced PARP cleavage, sustaining an antiproliferative activity explained by the inhibition of NF-κB signaling; in fact, in BC cell lysates, this compound inhibited the phosphorylation of IκBα, preventing IκBα from forming the NF-κB heterodimer (p65 and p50) necessary for the activation of the NF-κB axis, and inhibited the nuclear translocation of p65 (subunit of the NF-κB heterodimer) and its phosphorylation in the nucleus, leading to the inhibition of the transactivation of NF-κB target genes [238]. In a similar way, genistein treatment of MDA-MB-231 cells induced apoptosis in a dose- and time-dependent manner by inhibiting NF-κB activity via the Notch-1 signaling pathway. Moreover, genistein administration suppressed the expression of cyclin B1, Bcl-2, and Bcl-xL, possibly through the activation of NF-κB via the Notch-1 signaling axis [182]. Specifically, the phytoestrogen caused a dose-dependent reduction in the levels of mitogen-activated protein kinase 5 (MEK5), total ERK5, and phospho-ERK5 and a decrease in the levels of NF-κB/p65 nuclear protein, which are associated with inhibition of MDA-MB-231 cell proliferation and induction of apoptosis. Activation of the apoptotic process is confirmed by a dose-dependent increase in Bax protein levels and a decrease in Bcl-2 protein levels, as well as cleavage of caspase-3 and induction of caspase-3 activity [239]. Similarly, genistein has been shown to induce apoptotic cell death, as demonstrated by a decreased Bcl-2/Bax mRNA and protein ratio, in the MCF-7 cell line through a mechanism involving the inactivation of the type 1 insulin-like growth factor receptor (IGF-1R)/p-Akt signaling axis [240]. The p38-MAPK pathway drives cell proliferation and antiapoptosis, so the inhibition of this axis represents a good strategy for the promotion of cancer cell death [241]. In this regard, genistein induced apoptotic MCF-7 cell death through calpain and caspase-7 activation and PARP cleavage. In addition, administration of the phytoestrogen activated the apoptosis signaling kinase 1 (ASK1)-p38 mitogen-activated protein kinase cascades involving Ca^2+^ release from the endoplasmic reticulum, whereas no effect was observed for ERK1/2 [242]. Similarly, the isoflavone induces apoptosis in MCF-7 BC cells by activating Ca^2+^-dependent proapoptotic proteases, including mu-calpain and caspase-12, through the increase in intracellular Ca^2+^ concentration resulting from the depletion of endoplasmic reticulum Ca^2+^ stores [243].

The MAPK and AKT pathways are often constitutively activated in solid tumors; in contrast, the deletion of PTEN is one of the tumor suppressors often lacking in patients with advanced cancer [244]. In this regard, genistein-treated MCF-7 cells showed increased PTEN protein levels associated with decreased Akt phosphorylation. They increased p27 protein levels, whereas MAPK phosphorylation and cyclin D1 levels regulated by PTEN protein phosphatase activity were not altered, but their mRNA levels were slightly increased in phytoestrogen-stimulated cells [245]. Consistently, at low physiologically relevant concentrations, genistein inhibited cell survival and induced apoptosis in metastatic BC cells, MDA-MB-435 and Hs578t, while not affecting the survival of non-metastatic MCF-7 cells. In association with decreased cell viability and increased apoptosis, genistein decreases miR-155 and increases its proapoptotic targets including Forkhead box (Fox) subclass O3 (FOXO3), PTEN, casein kinase, and p27 [246]. A study by Dave et al. (2005) provided further insight into the PTEN-involving mechanism by which genistein promotes mammary epithelial cell death in vitro and in vivo. The mammary glands of young adult female rats exposed to diets containing casein (CAS) as the sole protein source supplemented with genistein or soy protein isolate (SPI+) showed increased apoptosis compared to rats fed a CAS diet without genistein. Increased mammary apoptosis in genistein and SPI+ rats was accompanied by increased PTEN expression. However, increased expression of the proapoptotic genes p21, Bax, and Bok was only observed in genistein-fed rats. Furthermore, genistein-induced apoptosis in MCF-7 cells was associated with increased PTEN expression and was abolished when PTEN expression was knocked down. MCF-7 cells treated with serum from genistein- or SPI(+)-fed rats showed increased apoptosis and increased levels of PTEN transcript [247].

In addition, genistein-induced apoptosis in MCF-7 cells was estrogen-independent and associated with dysregulation of the Bax/Bcl-2 ratio, downregulation of the antiapoptotic protein survivin, and induction of oxidative stress, as evidenced by decreased expression of the antioxidant enzymes copper–zinc superoxide dismutase (CuZnSOD), MnSOD, and thioredoxin reductases (TrxRs) and increased expression of glutathione peroxidase (GPx) [248]. Similarly, in MDA-MB-231 and MDA-MB-468 cells, genistein administration resulted in the downregulation of antiapoptotic Bcl-2, upregulation of proapoptotic Bax, and activation of caspase-3, leading to the induction of apoptotic death involving the mobilization of endogenous copper, as demonstrated by the fact that the copper-specific chelator neocuproine was able to almost completely reverse these gemistein-induced effects, whereas iron and zinc chelators were ineffective. Furthermore, the involvement of ROS production in genistein-induced apoptosis was demonstrated by its inhibition by ROS scavengers [249].

As specified above, genistein, as a phytoestrogen, can compete and/or interfere with the activity of Erα, and ERβ receptors can act as an estrogen agonist or antagonist in mammals by competing or interfering. In this regard, genistein was reported to have different effects against BC cells at different concentrations and in various cell types (ER-positive and ER-negative cells) [91,213,250].

For example, using proteomic and transcriptomic approaches, it has been shown that genistein has effects on gene and protein expression in T47D ERβ-positive cells, even in the presence of low levels of ERα, and that its final estrogenic effect on cells/tissues depends on both by the phenotype of receptors and the receptor subtype ratios within these cells/tissues. Precisely, this phenotype may be altered by exposure to genistein [251].

The necessity of ERα for estrogen-induced growth inhibition and apoptosis was confirmed by Obiorah et al. (2014) who showed that the phytoestrogen genistein induced endoplasmic reticulum stress, an inflammatory response leading to intrinsic and extrinsic apoptosis in a long-term estrogen-deprived BC cell line (MCF-7:5C) through an ERα-mediated mechanism. Genistein induced the endoplasmic reticulum stress (ERS) marker CHOP and the unfolded protein response (UPR) sensor inositol-requiring protein 1 alpha (IRE1α), whereas PCD activation was evidenced by upregulation of the apoptotic genes BCL2L11/BIM, tumor necrosis factor (TNF), FAS, and FADD [252]. Similarly, genistein at general physiological concentrations significantly reduced proliferation and increased apoptosis in primary BC cells, as evidenced by increases in apoptosis markers such as FADD, tBid, cyt c, caspase-8, and caspase-3. However, in the presence of E2, the ability of genistein to induce apoptotic effects in malignant breast cells ex vivo was completely abolished [253]. Furthermore, cellular responses have been shown to be triggered by different signaling mechanisms depending on the concentration of genistein. Physiological concentrations of this isoflafone (<10 μM) plus E2 induced apoptosis of MDA-MB-231 (ERβ-positive/ERα-negative) cells by dysregulating the Bax/Bcl-2 ratio and reducing the phosphorylation of ERK1/2, sustaining the antitumoral role of genistein against ERβ-positive/ERα-negative BC cells. At higher concentrations (10 to 100 μM), genistein reduced cell proliferation and increased apoptosis, both in the presence and absence of E2, without the involvement of Bax/Bcl-2 or the phosphorylation of ERK1/2 [254]. Pons et al. (2014) demonstrated that the outcome of genistein treatment depends on the ERα/ERβ ratio in BC cells. Co-administration of genistein with E2 induced cell proliferation and inhibited apoptosis in MCF-7 cells characterized by a high ERα/ERβ ratio, whereas in T47D cells with a low ERα/ERβ ratio, E2 and especially GEN exerted antiproliferative and proapoptotic effects as indicated by a significant decrease in the P-STAT3/STAT3 ratio [213].

In addition, genistein exhibited a biphasic effect on MCF-7 BC cell growth and ERα expression; in fact, low concentrations (<10 µM) of the phytoestrogen induced marked increases in proliferation and ERα expression, whereas high concentrations of the isoflavone caused inhibition of cell proliferation and apoptotic morphological features in treated cells. Furthermore, the expression of ERα and erbB2 was dose-dependently reduced by genistein for both SKBR3 and ZR-75-1 cells, and apoptotic changes were detected after exposure of ZR-75-1 cells to either daidzein or genistein at a concentration of 100 µM for 72 h [255]. Similarly, the isoflavone induced apoptosis in estrogen-sensitive MCF-7 cells at 50 and 100 µM, but estrogen interfered with the apoptotic activity of genistein at 50 µM. The estrogen-modulated increase in cyclin B1 levels was contrasted by co-treatment with genistein (100 μM), suggesting that the antiapoptotic regulation of cells by estrogen may involve cyclin B1 and that genistein exerted an estrogen-antagonistic action at a higher concentration [256].

The proapoptotic effect of genistein has been shown to be dependent on estrogen levels only in cultured cells, whereas in animal models estrogen levels did not affect the results. In detail, genistein administration induced apoptosis in MCF-7 cells in the absence of estrogen, as evidenced by DNA fragmentation, reduced levels of Bcl-2, and upregulated Bax protein. In the presence of estrogen, p21 and p53 protein expressions were upregulated by high concentrations of genistein. In female rats, the Bcl-2/Bax ratio was decreased by genistein treatment in the presence or absence of estrogen. These results indicate that the proapoptotic property of genistein may be strongly influenced by the concentration of estrogen in vitro, but this influence by estrogen is not evident in vivo [257]. Furthermore, the proliferation of MCF-7 cells was induced by both E2 alone and E2 plus genistein at concentrations ≥ 10^−6^ mol/L while apoptotic cell death was induced by GEN monotherapy in the same cell line [257]. The combination of genistein and E2 caused the increase in proliferating cell nuclear antigen, PI3K, and p-Akt and the inhibition of the increase in FADD, cyt c, truncated Bid, caspase-9, caspase-3, and ERβ [258]. Consistently, Lucki et al. (2011) have provided a mechanism to explain genistein’s ability to promote the proliferative response of MCF-7 cells via an estrogen-dependent ER-binding pathway, which predicts that genistein induces acid ceramidase (ASAH1) gene expression by activating a cell surface receptor-coupled G-protein (GRP30)-dependent pathway leading to the phosphorylation of ERK1/2 which in turn triggers the proliferative response of BC cells via an estrogen-dependent pathway. Moreover, ERK1/2 activation provokes the phosphorylation of ERα and the formation of a complex with ERα, Sp1, and SRC1 bound to the ASAH1 promoter. As a result of ASAH1 transcription, there is an increase in protein expression and enzymatic activity, which in turn leads to an increase in sphingosine 1-phosphate (S1P) production. By binding on cell surface S1P receptors, S1P can then be exported and activate proliferative pathways; in parallel, ASAH1 controls the expression of cyclin B2, thereby promoting mitosis and cell growth [259]. Instead, co-treatment of MCF-7 cells with different doses of E2 and genistein (10^−6^ M) did not result in antagonism of E2 activity and did not affect the proliferation rate. Co-treating MCF-7 cells with E2 and GEN resulted in a significantly increased inhibition of apoptosis, at least in combination with E2 (10^−10^ M) [260].

Moreover, the SUM1315MO2 cell model carrying the 185delAG BRCA1 mutation was markedly more sensitive to GEN than BRCA1 wild-type cell lines, probably due to the expression of ERβ, which is a major mechanism of physiological action of genistein, as already explained [261]. Genistein induced apoptosis more efficiently in BRCA1-mutant BC cell lines (HCC1937, SUM149, and SUM1315 cells) than in the MDA-MB-231 cell line, which harbors a functional wild-type BRCA1 gene. After genistein administration, all cells showed increased p21 protein levels and decreased Akt signaling, consistent with activation of the apoptotic process, except for MDA-MB-231 cells, in which mRNA and AKT protein levels were strongly increased and p21 protein levels slightly decreased, resulting in resistance to genistein [262].

The treatment with various concentrations of soya aglycone-rich extract (SARE) and flaxseed aglycone-rich extract (FSARE) caused higher caspase levels in MDA-MB-231 than in MCF-7 cells. Based on the docking score and binding energy, the best-fit protein target genistein was aldose reductase which showed a dose-dependent decrease in its expression after SARE administration [263]. In the study by Stocco et al. (2015), an extract of soy biotransformed by the fungus Aspergillus awamori was administered to estrogen-dependent (MCF-7) and non-estrogen-dependent (SKBR3) BC cell lines. The data suggested that the components of the extract, which was implemented in terms of the amount of isoflavones produced by the process of soy biotransformation, induced cell death by apoptosis and necrosis, mainly in MCF-7 cells, through a process responsive to caspase-3 activation involving, among other proapoptotic factors, Bad [264].

### 10.2. Prostate Cancer

In PC, similar effects were exhibited after the isoflavone administration: specifically, genistein administration to PC3 cells induced apoptotic death by increasing caspase-3 expression and activity. Cell growth was suppressed by the reduction of the p38 mitogen-activated protein kinase (p38MAPK) gene expression and protein level, while cell aggressiveness was strongly suppressed by the reduction in MMP-2 activity [265]. A similar result has been described by Kumi-Diaka et al. (2006); specifically, genistein exposure promoted the inhibition of cell growth through apoptotic cell death in both PC3 and LNCaP cells [266]. A significant dose- and time-dependent inhibition of MMP-2 expression levels in both cells was correlated with increased genistein concentrations [266]. In the study by Li et al. (2004), the effects of GEN on PC3 cells and experimental PC3 bone tumors created by injecting PC3 cells into human bone fragments previously implanted in severe combined immunodeficient (SCID) mice were evaluated. It was found that the isoflavone modulated the expression of genes involved in cell growth, apoptosis, and metastasis both in vitro and in vivo. Genistein administration caused the suppression of MMP-2, -11, -13, -14, and membrane-type matrix MMPs (MT-MMPs) and the upregulation of osteoprotegerin in PC3 bone tumors. Furthermore, MMP-9 expression was inhibited in PC3 cells in vitro and PC3 bone tumors in vivo after genistein administration [267]. Differently, the administration of genistein to PC3 cells resulted in the downregulation of the MDM2 oncogene mRNA, protein upregulation of p21, and induction of apoptosis. The downregulation of MDM2 protein is achieved by genistein-induced MDM2 ubiquitination, with the MDM2 promoter being important for the effects of genistein. Overexpression of MDM2 abolished genistein-induced apoptosis in vitro. These results were confirmed in vivo, as genistein inhibited tumor growth in PC3 xenografts [188]. By contrast, the effect of genistein in LNCaP and PC3 cancer cell lines was associated with the induction of apoptosis through the downregulation of PLK-1 protein and the upregulation of p21 [203]. Through epigenetic changes, genistein also affects gene expression patterns. In this regard, the modulation effects of genistein on DNA methylation led to the inhibition of cell growth and induction of apoptosis. In detail, the methylation profiles of 58 genes were altered by genistein administration in DU145 and LNCaP PC cells. In addition, the methylation frequencies of the mitotic arrest deficient 1-like protein 1 (MAD1L1), TNF receptor-associated factor 7 (TRAF7), lysine demethylase 4B (KDM4B), and human telomerase reverse transcriptase (hTERT) genes were remarkably modified by genistein treatment [268]. Furthermore, genistein has been shown to suppress growth and induce apoptotic death in PC3, DU145, and LNCaP cell lines in vitro by inducing the expression of aplasia Ras homology I (ARHI), a tumor suppressor gene downregulated in various malignancies including PC. ARHI was reported to upregulate the Hect domain and RLD5 (HERC5), CDNK1A, growth arrest, and DNA damage-inducible alpha (GADD45A) at transcriptional and protein levels. The effect of genistein was mediated by downregulation of miR-221 and miR-222 [269]. In addition, miR-574-3p was significantly upregulated in genistein-treated DU145 and PC3 PC cells compared to vehicle control. The expression of miR-574-3p was significantly lower in PC cell lines and clinical PC tissues than in normal prostate cells (RWPE-1) and adjacent normal tissues. miR-574-3p restoration induced apoptosis through reduction of Bcl-xL and activation of caspase-9 and caspase-3 [270]. Moreover, GEN administration modulated epigenetics and gene expression by altering histone acetylation through increased HAT1 protein levels resulting in increased H3K9 acetylation and increased SOX7, a Wnt inhibitory gene, and cell death promotion in PC3, DU145, ARCaP-E, ARCaP-M, and LNCaP cancer cell lines [271]. Similarly, genistein significantly induced apoptosis of DU145 cells, but no significant effect on apoptosis was observed in PC3 cells. lncRNA profiling showed that genistein modulated increased HOTAIR expression in castration-resistant PC cell lines compared to normal prostate cells. Indeed, PC cell growth and invasion were suppressed by HOTAIR knockdown (siRNA) in association with induction of apoptosis. miR-34a was also upregulated by GEN and directly targeted HOTAIR in both PC3 and DU145 PC cells [272]. Therefore, global gene expression patterns showed that maximal physiologically achievable concentrations of genistein (≤10 μM) had proliferative effects in PC3 cells by inducing activation of CDKs, MAPKs and small ribosomal subunit proteins (RPSKs), whereas high concentrations of genistein (>10 μM) appeared to modulate a different signaling axis leading to apoptotic cell death, specifically downregulating TGF-β by specifically decreasing SMAD 2/3, 4 in the downstream TGF-β signaling cascade [273]. This effect has also been shown in LNCaP cells where the isoflavones did not alter death receptor expression but significantly augmented TRAIL-induced disruption of mitochondrial membrane potential causing cytotoxic and apoptotic effects [274]. The oxidative-stress-promoting effect of GEN was confirmed in PC3 cells under 3D culture conditions, where a concentration of 480 μM of the isoflavone reduced cancer cell viability by inducing apoptosis through a non-mitochondrial pathway; furthermore, genistein reduced the production of cellular nitric oxide (NO) and increased catalase and glutathione production [275]. Furthermore, genistein inhibited the growth of DU145 and LNCaP cancer cells, leading to the death of such cells, by inducing ROS production and interfering with the expression of the two copper transporter genes, CTR1 and ATP7A. The copper chelator neocuproine reversed this effect, highlighting the role of copper in isoflavone-induced cytotoxicity [276].

In an animal model study by Nakamura et al. (2011), genistein has been shown to promote metastatic activity in a patient-derived PC (LTL163a) xenograft NOD-SCID mouse model, where increased lymph node and secondary organ metastases were demonstrated in genistein-treated mice compared to controls. More proliferating and fewer apoptotic cancer cells were found in paraffin sections from genistein-treated groups compared to the untreated group. Immunoblotting data showed that the activities of tyrosine kinases, EGFR and its downstream mediator Src, were increased in the genistein-treated groups [106]. Furthermore, 18 months of consumption of 19.2 g/day of whole soy protein isolate containing 24 mg genistein by middle-aged to older males reduced circulating testosterone and sex-hormone-binding globulin (SHBG) but not free testosterone SHBG compared with the casein-based placebo. Indeed, serum concentrations of estradiol, VEGF, insulin growth factor-1 (IGF-1), insulin-like growth-factor-binding protein-3 (IGFBP-3), IGF-1/IGFBP-3 ratio, soluble Fas, Fas-ligand, and sFas/Fas-ligand ratio were not affected, and the apoptotic process was not induced [277]. Similar effects have been described using natural mixtures of isoflavones with high genistein content as analyzed below. For example, an isoflavone mixture (83.3% genistein, 14.6% daidzein, and 0.26% glycitein) inhibited the phosphorylation of Akt and FOXO3a, regulated the phosphorylation of Src, and increased the expression of glycogen synthase kinase-3β (GSK-3β), leading to the downregulation of androgen receptor (AR) and its target gene prostate-specific antigen (PSA) with the result of induction of apoptosis in both androgen-sensitive and -insensitive PC cells [278]. Furthermore, in LNCaP and PC3 cells, soy extract was shown to be more effective than soy isoflavones, inducing cell cycle arrest and apoptosis. Soybean extract induced cell cycle arrest, activated caspases, and increased Bax via NF-κB-independent routes [279]. Genistein combined polysaccharide (GCP), a dietary supplement containing the isoflavones genistein, daidzein, and glycitein, mediated growth inhibition and apoptosis of PC cells by multiple mechanisms, including molecular mimicry of androgen ablation (via AR downregulation) and by an AR-independent proapoptotic signal (mTOR inhibition) [280]. Furthermore, GCP and perifosine were able to induce growth arrest in LNCaP (androgen sensitive), LNCaP-R273H, C4-2, Cds1, and PC3 (androgen insensitive) PC cell lines, associated with increased inhibition of Akt activity and induction of p21 expression. Only LNCaP cells showed increased apoptosis, further enhanced after AR knockdown [281]. Similarly, GCP potentiated the activity of the AR antagonist bicalutamide, the antimicrotubule taxane docetaxel, and the Src kinase inhibitor pp2 in LNCaP, CWR22Rv1, PC3, and LNCaP-R273H cell lines, causing growth inhibition and increased apoptosis. In more detail, the combination of GCP and bicalutamide had enhanced activity in both the LNCaP and LNCaP-R273H lines whereas LNCaP cells exhibited increased apoptosis when docetaxel administration was followed by GCP [282]. Similar results were reported by Bemis et al. (2004), demonstrating that GCP inhibited LNCaP and PC3 cell growth by inducing apoptosis in LNCaP cells but not in PC3 cells. GCP induced p27 and p53 protein expression only in LNCaP cells and suppressed phosphorylated Akt in both cell lines. Furthermore, a 2% GCP-supplemented diet delayed tumor growth and increased apoptosis in LNCaP xenograft tumor-bearing immunodeficient mice [283]. A clinical trial in PC patients showed that lycopene and soy isoflavones delayed the progression of both hormone-refractory and hormone-sensitive PC; however, no additive effect between the two compounds was demonstrated [284]. The main targets of genistein in the activation of apoptotic death in prostate cancer and BC are summarized in Table 2.

While the aforementioned studies focus on genistein’s antitumor activity in hormone-dependent tumors, particularly PC and BC, evidence also suggests that isoflavones may exhibit antitumor activity in other solid tumors [215,285,286]. Furthermore, the antitumor activity of genistein against various hematological tumors has also been confirmed by several studies [287,288,289].

### 10.3. Combinatorial Strategy

Combinations of genistein agents with different molecular mechanisms of action are promising, as they may be more effective and have fewer systemic toxicities. More specifically, synergism and the interaction of the isoflavone with different types of chemotherapeutics or natural bioactive have been studied. Genistein, administered alone or in combination with doxorubicin, induced apoptotic death in the resistant derivative cell line MCF-7/Adr by downregulating Her2/neu mRNA expression in a dose-dependent manner, but had no effect on multidrug resistance 1 (mdr-1) mRNA expression. Her2, a member of the EGFR family, is a membrane tyrosine kinase (TK) that is overexpressed in several types of cancer, where it leads to the activation of downstream oncogenic cascades such as the Ras/MAPK and PI3K/Akt pathways [290]. Furthermore, pretreatment of PC3 and MDA-MB-231 with genistein in vitro and in vivo inactivates NF-κB, thereby sensitizing cancer cells to chemotherapeutic growth inhibition and apoptosis. Genistein at 15 to 30 μM in combination with 100 to 500 nM of cisplatin, 0.5 to 2 nM of docetaxel, or 50 ng/mL of doxorubicin resulted in a significantly greater inhibition of PC3 (PC), MDA-MB-231 (BC) cell growth, and induction of apoptosis compared to any of these agents alone. In addition, NF-κB activity was significantly increased within 2 h of treatment with cisplatin and docetaxel, and the NF-κB-inducing activity of these agents was completely abolished in cells pretreated with genistein [291]. These results were further confirmed by animal experiments performed in vivo in PC3 tumors established in SCID mice treated with doxacetal, which demonstrated that a specific target (NF-κB) was affected in vivo [291]. Consistently, genistein was shown to enhance the antitumor effects of cisplatin via mitochondria-mediated apoptosis in ERα-deficient MDA-MB-231 cells (ERα−/ERβ+) but not in normal MDA-MB-231 cells. Indeed, Bax/Bcl-2 and p21 expression ratios appeared even more sensitive to cisplatin or genistein + cisplatin treatment, suggesting that p53-independent regulation of Bax/Bcl-2 and p21 may play a role in cisplatin’s antitumor effects and genistein’s procisplatin bioactivity [292]. Conversely, a study by Hu et al. (2014) showed that genistein counteracted the antitumor potential of cisplatin only in the absence of E2 through the modulation of apoptosis and proliferation of MCF-7 cells. Through a mechanism mediated by ERα, the presence of E2 allowed abolition of the anticisplatin effect of genistein as shown by the increase in Bax/Bcl-xL [293]. These results were confirmed in vivo where oral administration of genistein inhibited the antitumor effects of cisplatin treatment in ovariectomized BC EMT6 xenograft tumor mouse models. At the molecular level, the Bax/Bcl-2-associated mitochondria-dependent apoptosis was blocked by genistein [294]. Moreover, in cells with a high ERα/ERβ ratio (MCF-7), genistein affects the efficacy of chemotherapeutic agents by reducing ROS production and apoptosis induction (in cells treated with cisplatin) or autophagic cell death (in cells treated with tamoxifen), whereas in cells with a low ERα/ERβ ratio (T47D and MCF-7 + ERβ), the effect of genistein administration is less pronounced [295].

Whole-genome expression analysis showed that the combination of genistein and the histone deacetylase inhibitor vorinostat induced apoptotic cell death by decreasing baculoviral IAP repeat-containing protein 7 (BIRC7/Livin), transforming growth factor beta-1-induced transcript 1 protein (TGFB1I1/ARA55), hairy and enhancer of split-1 (HES1), and Snail family transcriptional repressor 2 (SLUG), which are involved in the TNF-NF-κB and androgen signaling pathways in PC3, DU145, ARCaP-E, ARCaP-M, and LNCaP cancer cell lines [271].

A mechanism opposing the action of specific drugs has been shown for genistein in several cancer cell lines; in fact, the isoflavone counteracted the induction of apoptosis by the chemotherapeutic tubulin-binding compounds paclitaxel or vincristine by reducing Bcl-2 phosphorylation and suppressing cyclin B1 and CDC2 kinase expression without altering Bax protein expression in MCF-7 and MDA-MB-231 cells [296].

Conversely, genistein enhanced the effect of the chemotherapeutic cabazitaxel by promoting both the upregulation of the proapoptotic protein Bax and the activation of apoptotic signaling in mCRPC cells. In a PC3 luciferase xenograft model, the combined treatment significantly delayed mCRPC growth compared to vehicle control or monotherapy. Tissue staining confirmed the in vivo effect of genistein on Bax induction and apoptosis activation [297]. In addition, both genistein and topotecan induce cell death in LNCaP cells, with the combination of genistein and topotecan being significantly more effective in reducing the viability of LNCaP cells than either genistein or topotecan alone. Cell death was primarily apoptotic via activation of caspase-3 and -9, which are involved in the intrinsic pathway, and associated with increased ROS generation with genistein–topotecan combination treatment [298].

Genistein may affect estrogen-modulated pathways by antagonizing or agonizing the action of estrogen pathway modulators. The combination of tamoxifen, a drug used for the initial treatment and prevention of estrogen-receptor-positive (ER+) breast tumors, and genistein synergized in vitro inhibition of the growth of ER+/HER2-overexpressing BT-474 human BC cells. Co-administration of genistein and tamoxifen induced apoptotic cell death as evidenced by an increase in DNA fragmentation and downregulation of mRNA and protein expression of survivin, one of the apoptotic effectors, and downregulation of EGFR, HER2, and ER expression [214]. Moreover, the combination of genistein and centchroman (CC), a selective ER modulator used as a non-steroidal oral contraceptive, caused a ROS-dependent induction of apoptosis in MCF-7 and MDA-MB-231 cells, as evidenced by increased Bax/Bcl-2 ratio, activation of caspases-3, -7, and -9, and PARP cleavage. The PCD is at least partly achieved by strong suppression of the PI3K/Akt/NF-κB pathway after genistein and CC administration, as shown by decreased phosphorylation of PI3K and Akt and decreased levels of NF-κB in both cell lines and decreased phosphorylated mTOR found only in MCF-7 cells. These data were confirmed by in vivo evidence, where the combination therapy of CC and genistein was well tolerated and induced a significant reduction in tumor growth in comparison to the single therapies in the mouse 4Ti breast tumor model [299]. The co-administration of the steroid hormone precursor 1,25(OH)2D3 and the phytoestrogen genistein exerted a synergistic effect on apoptosis of MCF-7 and MDA-MB-231 BC cells as evidenced by an increase in BAX and CASP3 gene expression and downregulation of the BCL-2 gene compared to the single treatment [187]. Similarly, the sensitivity of PC DU145 cells to the growth inhibitory effects of 1alpha-25-dihydroxyvitamin D3 (1,25(OH)2D3) is increased by co-treatment with genistein. The mechanism underlying the effect of genistein is a direct non-competitive inhibition of mitochondrial CYP24 enzyme activity. Genistein potentiated the action of 1,25(OH)2D3 by directly inhibiting CYP24 enzyme activity and prolonging the half-life of 1,25(OH)2D3 with upregulation of the vitamin D receptor (VDR) both at the mRNA and protein levels [300].

The combination of genistein and the cholesterol-lowering agent 2-hydroxypropyl-beta-cyclodextrin (HPCD) resulted in greater inhibition of LNCaP PC cell growth compared to genistein treatment alone. Apoptosis induction was demonstrated by PARP cleavage and caspase-3 activation. In addition, the EGFR-mediated phosphorylation cascade of Akt, GSK-3beta, and p70S6k was significantly inhibited by the combination treatment, and downregulation of AR was detected in the lipid raft microdomain [301]. A non-toxic dose of terazosin synergized the antitumor activity of genistein on DU145 human PC cells. The combination of genistein and terazosin caused a more significant decrease in Bcl-XL levels in DU145 cells compared to genistein treatment alone [302].

A mechanism of increased cell killing by combined genistein and radiation treatment has been proposed to be triggered by inhibition of NF-κB, leading to altered transcription of cell cycle regulatory proteins such as cyclin B and/or p21WAF1/Cip1, thereby promoting apoptotic cell death. Increased apoptotic cell death was confirmed by observing significantly increased expression levels of cleaved PARP protein in cells treated with genistein and irradiation compared to each modality alone, demonstrating increased apoptotic cell death [202]. The sensitivity of MCF-7 and MDA-MB-231 cells to genistein increased after cell X-ray irradiation leading to cell apoptotic death as evidenced by upregulation of Bax and protein 73 (p73) protein levels and downregulation of Bcl-2 protein expression. Of note, p73 is a tumor suppressor protein related to the tumor protein p53 [196]. Compared to GEN or radiation alone, the combination of genistein and radiation produced an increase in giant cells, apoptosis, inflammatory cells, and fibrosis with decreased tumor cell proliferation consistent with increased tumor cell destruction. Genistein alone increased the size of heavily infiltrated lymph nodes and prostate tumors were larger and had more necrotic cells, apoptotic cells, and giant cells than control tumors observed after radiation and GEN treatment [303]. The combination of genistein and ionizing radiation (IR) induced apoptosis, prolonged cell cycle arrest, and disrupted damage repair in DU145 PC cells [304].

The combination of genistein and photodynamic therapy with hypericin was effective in inducing apoptosis in MCF-7 and MDA-MB-231 breast adenocarcinoma cells in particular. This process was associated with the increment in Bax/Bcl-2 ratio in both cell lines and suppression of Akt and Erk1/2 phosphorylation induced by photoactivated hypericin in MCF-7 cells, whereas Akt and Erk1/2 phosphorylation, which was not stimulated by photodynamic therapy with hypericin in MDA-MB-231 cells, was effectively suppressed in combinatory treatment [305].

A synergistic effect has been described between genistein and specific inhibitors of enzymes involved in important cellular pathways as described below. Treatment with genistein alone or in combination with the TK inhibitor (tyrphostin) AG1024 increased the radiosensitivity of PC cells (DU145) to X-irradiation by cell cycle arrest and induction of apoptosis [306]. Although genistein hurt the efficacy of the aromatase inhibitors letrozole (Let) and anastrozole (Ana), its combination with the aromatase inhibitor Exe potentiated the antiproliferative and apoptotic effect of the single treatment in sensitive (MCF-7-aro) and resistant (LTEDaro) BC cells evidenced by the increase in the ratio of cleaved PARP/PARP [307]. Co-administration of genistein and the kinesin spindle protein (KSP) inhibitor SB715992 resulted in significantly greater inhibition of PC3 cell growth and induction of apoptosis compared to the effects of either agent alone [308].

The ability of genistein to epigenetically modulate aberrant gene expression patterns in BC cells was confirmed by Lubecka et al. (2018) who showed that exposure of MCF-7 and MDA-MB-231 cells to the combination of genistein and clofarabine (2-chloro-2′-fluoro-2′-deoxyarabinosyladenine, ClF), a second-generation 2′-deoxyadenosine analog capable of regulating epigenetic processes, resulted in strong upregulation and activation of DNA-methylation-silenced tumor suppressors, including PTEN, retinoic acid receptor beta (RARB), and CDKN1A, and consequent activation of apoptosis [309]. Another proposed strategy has combined genistein and CD36 siRNA-loaded self-assembled DNA nanoprisms (NP-siCD36) as a treatment for TNBC cells (MDA-MB-231). CD36 is a glycoprotein involved in the transport of fatty acids. Both genistein and NP-siCD36 have a more potent effect on CD36 and phospho-p38 suppression compared to the individual monotherapies [310]. Genistein mediates apoptosis via the caspase-3 cascade in DU145 cells. Combined treatment with survivin RNA interference (RNAi) and varying concentrations of genistein showed a stronger inducible apoptotic effect on DU145 prostate cells [311].

Administration of genistein and the ortho-naphthoquinone beta-lapachone (bLap) promoted apoptosis in PC3 cells by targeting mainly caspase-3 (CPP32) and NAD(P)H quinone oxidoreductase (NQO1), respectively [312].

Several natural bioactive agents have been shown to exert a synergistic antitumoral effect with genistein. For example, daidzein and genistein showed a synergistic effect in inhibiting cell proliferation and inducing apoptosis in early-stage androgen-dependent PC cells (LNCaP) and bone-metastatic LNCaP-derived PC cells (C4-2B cells). In more detail, 25 or 50 μM daidzein/50 μM genistein significantly increased the apoptotic effects on C4-2B cells, although they had no effect when used alone [313]. Furthermore, apoptosis induction was achieved by the combination of genistein and equol, a metabolite of daidzein, in MCF-7 cells but not in SK-BR-3 cells, as evidenced by the increase in the Bax/Bcl-xL expression ratio, without affecting the activities of Akt and mTOR [314]. In addition, genistein-induced apoptosis in MCF-7 BC cells, and combined treatment with genistein and pomegranate extracts enhanced apoptosis compared to a single treatment [315]. Moreover, the combination of genistein and sulforaphane, a natural compound found in cruciferous vegetables, exerted synergistic effects in reducing the cellular viability of MDA-MB-231 and MCF-7 BC cell lines. The mechanism involved the downregulation of Krüppel-like factor 4 (KLF4), an oncogene-acting gene in BC, and HDAC2 and HDAC3 activity, whose inhibitor-mediated inhibition is dependent on KLF4. In addition, hTERT, which is also overactivated by KLF4 in BC, was downregulated by the combination of genistein and sulforaphane [316]. In another study, the combination of sulforaphane, sodium butyrate, a short-chain fatty acid, and genistein exerted a synergistic induction of the apoptotic pathway in MDA-MB-231 and MCF-7 BC cells compared to the compounds administered alone. The tri-combinatorial treatment caused genome-wide epigenetic modification through the downregulation of DNA methyltransferases, histone deacetylases, and histone methylases and the upregulation of histone acetyltransferases [317]. Selenite and genistein have shown synergistic effects on apoptosis, cell cycle arrest, and associated signaling pathways in p53-expressing LNCaP and p53-null PC3 PC cells. Selenite or genistein treatment alone increased p53 protein levels only in LNCaP cells, whereas p21(waf1) and Bax were upregulated in both cell lines. In addition, only genistein inhibited AKT phosphorylation [201].

The target pathways hit by the combinatory treatments with genistein in breast and prostate cancers are summarized in Table 3.

### 10.4. Autophagy and Ferroptosis

Genistein has been shown to induce other types of PCD (i.e., autophagy and ferroptosis). In MCF-7 cells, genistein has been shown to trigger apoptosis and autophagy through the induction of oxidative stress, as evidenced by the reduction of antioxidant enzymes and upregulation of GPx expression. Autophagy was demonstrated by the presence of LC3-positive puncta characteristic of autophagosome formation in treated cells [248].

The combination of genistein and daidzein induced ferroptotic death only in MDA-MB-231 cells, whereas this mechanism was not responsible for genistein- or daidzein-induced cell death in MCF-7 cells. Furthermore, both phytochemicals induced biochemical markers of ferroptosis, including lipid peroxidation and iron ion levels, and decreased GSH/GSSG levels. The mRNA expression levels of the most important antiferroptosis genes, Gpx4 and Fsp-1, were reduced by treatment with both phytochemicals. Pretreatment of MDA-MB-231 cells with ferrostatin-1 reversed the viability of these cells, confirming the activation of the ferroptotic process [318].

Taken together, this experimental evidence provides pivotal details about the prodeath action of genistein on cancer in both in vitro and in vivo circumstances.

## 11. Conclusions and Future Perspectives

Cancer is nowadays a major public health problem affecting an increasing number of people and is still the second leading cause of death worldwide [319]. Over the last 10 years, considerable evidence has confirmed that several natural compounds found in many edible plants have chemopreventive and antitumoral properties, with fewer side effects than current pharmacological strategies, which are often expensive, unspecific, and even significantly toxic [320,321,322,323]. In addition, a healthy and vegetable-rich diet is a valuable tool in the prevention, management, and treatment of many chronic and degenerative diseases, including cancer [324,325]. Consistently, several epidemiological studies have shown an association between a soy-rich diet and cancer prevention, attributed to the presence of genistein, a biologically active isoflavone [215,326,327,328]. Genistein has been shown to modulate a wide range of signaling pathways commonly altered in cancer, acting mainly by inhibiting angiogenesis and metastasis, affecting both EMT and the invasive potential of CSCs and altering the cell cycle and programmed cell death. The antioxidant and anti-inflammatory activities of this isoflavone are also known [19]. This review summarizes the antitumor activities of genistein in vitro and in vivo, emphasizing, in detail, the currently known mechanism of action. Isoflavones show structural similarity and biological activity to estrogen hormones and are also known as phytoestrogens. They are therefore able to compete with E2 for binding to the ERs and exert agonistic or antagonistic activity. In hormone-dependent tumors such as breast and prostate cancers, the effects of genistein can be either antiapoptotic and proliferative or proapoptotic and inhibitory to cell growth, depending on the concentration of phytoestrogen and the target cell type. The same synergistic or agonistic effect has been shown in studies in which genistein has been administered with different types of chemotherapeutic agents and natural compounds with known antitumor activity. In this case, again, the outcome of the combination was dependent on the concentration of the bioactive compound and the characteristics of the target cells. Chemically, the highly hydrophobic nature of genistein, and consequently its poor bioavailability, limits its biological applications. For this reason, current research tends to investigate and identify new methods and approaches to increase the bioavailability of genistein and its delivery to specific cells via nanovectors such as nanovesicles and liposomes [329,330,331,332,333]. Therefore, further long-term prospective studies and clinical trials are needed to fully understand the mechanism of action of this isoflavone in different cellular and pathological conditions, with a perspective of a possible wider use of genistein in cancer management.

## Figures and Tables

**Table 1:** Foods with the highest genistein content [32].

Source	Content (mg/100 g)
Soybeans seeds	5.56–267.2
Miso	33.69–67.20
Natto	21.52–59.37
Tempeh	1.11–112.21
Pistachio nuts	0.10–3.40
Chickpeas	0.069–0.214
Peanuts	0.02–0.39
Lentils	0.00–0.36
Parsley	0.057
Almonds	0.00–0.01

**Table 2:** Cell death induced by genistein in breast and prostate cancers.

	Genistein-Treated Cells/Animals	Molecular Pathway/Protein	Effect	Ref.
Breast cancer	MCF-7 overexpressing Bcl-2	↑ Bcl-2; ↑ p85; ↑ cyt *c*	↑ apoptosis	[229]
	MCF-7	↓ Bcl-2; ↑ Bax; ERα	↑ apoptosis	[230]
	MDA-MB-231	↑ p-p53; ↑ p21; ↓ Bcl-xL; ↓ cyclin B1	↑ apoptosis	[231]
	MCF-7	↓ Bcl-2/Bax	↑ apoptosis	[233]
	MDA-MB-231 and SKBR3	↓S kp2; ↑ p21; ↑ p27	↑ apoptosis	[197]
	MCF-7	↓ MDM2; ↑ p21	↑ apoptosis	[188]
	MCF-7	↓ p-Akt; ↓ HOTAIR	↑ apoptosis	[234]
	MCF-7-caspase-3 and T47D	↓ CIP2A; ↑ caspase-3; ↑ c-PARP	↑ apoptosis	[232]
	MCF-7	↓ PI3K; ↓ Akt; ↑ Fas; ↑ FADD; ↑ cyt c; ↑ t-Bid; ↑ caspase-9; ↑ caspase-3;	↑ apoptosis	[236]
	MCF-7 overexpressing HER2	↑ p53; ↑ Fas receptor; ↑ c-PARP; ↑ caspase-9; ↓ p-IκBα; ↓ NF-κB	↑ apoptosis	[238]
	MDA-MB-231	↓ NF-κB; ↓ Notch-1; ↓ cyclin B1, ↓ Bcl-2; ↓ Bcl-xL,	↑ apoptosis	[198]
	MDA-MB-231	↓ MEK5; ↓ ERK5; ↓ p-ERK5; ↓ NF-κB/p65; ↑ Bax; ↓ Bcl-2; ↑ caspase-3	↑ apoptosis	[199]
	MCF-7	↓ Bcl-2/Bax; ↓ IGF-1R/p-Akt	↑ apoptosis	[240]
	MCF-7	↑ calpain; ↑ Ca^++^; ↑ caspase-7; ↑ c-PARP; ↑ ASK1-p38 MAPK	↑ apoptosis	[242]
	MCF-7	↑ Ca^++^; ↑ mu-calpain; ↑ caspase-12	↑ apoptosis	[243]
	MCF-7	↑ PTEN; ↓ p-Akt; ↑ p27	↑ apoptosis	[245]
	MDA-MB-435 and Hs578t	↓ miR-155; ↑ FOXO3; ↑ PTEN; ↑ casein kinase; ↑ p27	↑ apoptosis	[246]
	Rats MCF-7	↑ PTEN; ↑ p21; ↑ Bax; ↑ Bok; ↑ PTEN	↑ apoptosis ↑ apoptosis	[247]
	MCF-7	↓ Bcl-2/Bax; ↓ survivin; ↓ CuZnSOD; ↓ MnSOD; ↓ TrxR; ↑ GPx	↑ apoptosis	[248]
	MDA-MB-231 and MDA-MB-468	↓ Bcl-2; ↑ Bax;↑ caspase-3; ↑ ROS; ↑ Cu	↑ apoptosis	[249]
	T47D	↑ IRE1α; ↑ CHOP; ↑ Bim; ↑ TNF; ↑ FAS; ↑ FADD	↑ apoptosis	[252]
	Primary breast cancer cells +17β-estradiol	↑ FADD; ↑ tBid; ↑ cyt c; ↑ caspase-8; ↑ caspase-3	↑ apoptosis ↓ apoptosis	[253]
	MDA-MB-231 GEN (<10 μM) + 17 β-estradiol GEN (10–100 μM) ± 17β-estradiol	↓ Bax/Bcl-2; ↓ p-ERK1/2	↑ apoptosis ↑ apoptosis	[254]
	MCF-7 + 17β -estradiol T47D + 17β -estradiol	↓ p-STAT3/STAT3	↑ proliferation ↑ apoptosis	[213]
	MCF-7 SK; BR 3 and ZR-75-1 GEN (<10 μM) GEN (10–100 μM)	↑ ERα ↓ ERα and erbB2	↑ proliferation ↑ apoptosis	[255]
	MCF-7 GEN (50 μM) + 17β -estradiol GEN (100 μM) + 17β -estradiol	↑ Cyclin B1 ↓ Cyclin B1	↓ apoptosis ↑ apoptosis	[256]
	MCF-7 +17 β-estradiol Female rats ±17 β-estradiol	↓ Bcl-2; ↑ Bax; ↑ p21; ↑ p53 ↓ Bcl-2; ↑ Bax;	↑ apoptosis No apoptosis ↓ tumor growth	[257]
	MCF-7 +17β -estradiol	↑ cell nuclear antigen; ↑ PI3K; ↑ p-Akt; ↓ FADD; ↓ cyt c; ↓ tBid; ↓ caspase-9; ↓ caspase-3; ↓ ERβ	↓ apoptosis	[258]
	MCF-7 20 nm GEN or 5 nm E_2_	↑ ASAH1; ↑ GRP30; ↑ p-ERK1/2; ↑ p-Erα; ↑ S1P	↑ proliferation	[259]
	MCF-7 +17β -estradiol [10^−10^ M]		↓ apoptosis	[260]
	SUM1315MO2 (185delAG BRCA1)	↓ ERβ	↑ apoptosis	[261]
	HCC1937, SUM149, SUM1315 MDA-MB-231	↑ p21; ↓ Akt ↑ Akt; ↓ p21	↑ apoptosis ↓ apoptosis	[262]
Prostate cancer	PC3	↑ caspase-3; ↓ p38MAPK; ↓ MMP-2	↑ apoptosis	[265]
	PC3 and LNCaP	↓ MMP-2	↑ apoptosis	[266]
	PC3 PC3-SCID mouse	↓ MMP-2; ↓ MMP-11; ↓ MMP-13; ↓ MMP-14; ↓ MT-MMP; ↑ osteoprotegerin ↓ MMP-9n	↑ apoptosis ↑ apoptosis	[267]
	PC3 PC3 xenograft	↓ MMP-2; ↑ p21	↑ apoptosis ↓ tumor growth	[188]
	LNCaP and PC3	↓ PLK-1; ↑ p21	↑ apoptosis	[203]
	PC3, DU145 and LNCap	↑ ARHI; ↑ HERC5; ↑ CDNK1A; ↑ GADD45A; ↓ miR-221; ↓ miR-222	↑ apoptosis	[269]
	DU145 and PC3	↑ miR-574-3p; ↓ Bcl-xL; ↑ caspase-9; ↑ caspase-3	↑ apoptosis	[270]
	PC3, DU145, ARCaP-E, ARCaP-M, and LNCaP	↑ HAT1; ↑ H3K9 acetylation; ↑ SOX7	↑ apoptosis	[281]
	DU145 PC3	↑ HOTAIR; ↑ miR-34a ↑ HOTAIR; ↑ miR-34a	↑ apoptosis no change	[272]
	PC3 GEN (≤10 μM) GEN (>10 μM)	↑ CDKs, ↑ MAPKs; ↑ RPSKs, ↓ TGF-β; ↓ SMAD 2/3,4	↑ proliferation ↑ apoptosis	[273]
	LNCaP	↑ TRAIL; ↓ MMP	↑ apoptosis	[274]
	3D culture PC3	↓ NO; ↑ catalase; ↑ GSH	↑ apoptosis	[275]
	DU145 and LNCaP	↑ ROS; ↑ CTR1; ↑ ATP7A	↑ apoptosis	[276]
	LTL163a) xenograft NOD-SCID mouse	↑ tyrosine kinases; ↑ EGFR; ↑ Src	↑ proliferation ↓ apoptosis	[106]

Akt/PKB, phospho-protein kinase B; ARHI/DIRAS3, Aplasia Ras Homology I; ASAH1, N-Acylsphingosine Amidohydrolase 1; ASK1, Apoptosis signal-regulating kinase 1; ATP7A, ATPase Copper Transporting Alpha; Bax, BCL2 Associated X; Bcl-2, B-cell lymphoma-2; Bcl-xL, B-cell lymphoma extra-large; Bid, BH3-interacting-domain death agonist; Bim, Bcl-2 Interacting Mediator of cell death; Bok, Bcl-2-related ovarian killer; CDK, cyclin-dependent kinase; CDNK1A, cyclin-dependent kinase inhibitor 1A; CHOP, C/EBP homologous protein; CIP2A, Cellular Inhibitor Of PP2A; Cyt *c*, cytochrome *c*; c-PARP, cleaved-Poly-ADP ribose polymerase; CTR1, High affinity copper uptake protein 1; EGFR, epidermal growth factor receptor; ERα, estrogen receptor α; ERβ, estrogen receptor β; ErbB2, c-Neu or human EGF receptor 2; ERK, extracellular signal-regulated protein kinases; FADD, FAS-associated protein with death domain; Fas, apoptosis stimulating fragment; FOXO3, forkhead box O3; GPx, Glutathione peroxidase; GSH, glutathione; GADD45A, growth arrest and DNA damage-inducible, alpha; GRP30, G protein-coupled receptor 30; HAT1, histone acetyltransferase 1; HERC5, Hect domain and RLD5; HOTAIR, HOX Antisense Intergenic RNA; IRE1α, Inositol-requiring transmembrane kinase/endoribonuclease 1α; MAPK, mitogen-activated protein kinase; MDM2, Mouse Double Minute 2; MMP- Metalloprotease-; NF-κB, nuclear factor kappa-B; NO, nitric oxide; p-Akt, phospho-protein kinase B; PARP, Poly-ADP ribose polymerase; p-ERK, phospho-extracellular signal-regulated protein kinases; PI3K, phosphoinositide 3-kinase; p-IκBα, phosphor-nuclear factor of kappa light polypeptide gen enhancer in B-cells inhibitor alpha; PLK-1, polo-like kinase 1;PP2A, Protein phosphatase 2; p-STAT, phospho-signal transducer and activator of transcription PTEN, Phosphatase and tensin homolog; ROS, reactive oxygen species; RPSK, Small ribosomal subunit protein uS11SMAD, Suppressor of Mothers against Decapentaplegic; Skp2, S-Phase Kinase Associated Protein 2; Src, Proto-Oncogene Tyrosine-Protein Kinase; STAT, signal transducer and activator of transcription SOD, superoxide dismutase; SOX7, SRY-Box Transcription Factor 7; S1P, Sphingosine 1-phosphate; t-Bid, truncated Bid; TGF-β Transforming growth factor-beta; TNF-α, tumor necrosis factor alpha; TRAIL, TNF-related apoptosis-inducing ligand; Trx, thioredoxin; ↑ = increase; ↓ = decrease.

**Table 3:** Combinatory treatment with genistein in breast and prostate cancers.

Genistein-Treated Cells/Animals	Combination with Genistein	Molecular Pathway/Protein	Effect	Ref.
MCF-7/Adr	doxorubicin	↓ Her2/neu	↑ apoptosis	[290]
PC3 and MDA-MB-231 PC3 SCID mice	cisplatin, docetaxel, or doxorubicin docetaxel	↓ NF-κB	↑ apoptosis ↑ growth inhibition	[291]
ERα- MDA-MB-231	cisplatin	↓ Bcl-2/Bax; ↑ p21	↑ apoptosis	[292]
MCF-7s	cisplatin (-17β-estradiol)	↑ Bax/Bcl-xl	↓ apoptosis	[293]
EMT6 xenograft mice	Cisplatin	↑ Bcl-2/Bax	↓ apoptosis	[294]
MCF-7 (high ERa/Erb) T47D (low ERa/Erb) MCF7 + ERβ	cisplatin	↓ ROS	↓ apoptosis ↑ apoptosis ↑ apoptosis	[295]
PC3, DU145, ARCaP-E, ARCaP-M, and LNCaP	vorinostat	↓ BIRC7/Livin; ↓ TGFB1I1/ARA55; ↓ HES1; ↓ SLUG	↑ apoptosis	[271]
MCF-7 and MDA-MB-231	paclitaxel or vincristine	↓ p-Bcl-2; ↓ cyclin B1; ↓ CDC2	↓ apoptosis	[296]
mCRPC	cabazitaxel	↑ Bax	↑ apoptosis	[297]
LNCaP	topotecan	↑ caspase-3; ↑ caspase-9; ↑ ROS	↑ apoptosis	[298]
BT-474-overexpressing ER+/HER2	tamoxifen	↓ survivin; ↓ EGFR; ↓ Her2; ↓ ERα	↑ apoptosis	[214]
MCF-7 and MDA-MB-231 mouse 4Ti breast tumor	centchroman	↑ ROS; ↑ Bax/Bcl-2; ↑ caspase-3; ↑ caspase-7; ↑ caspase-9; ↑ c-PARP; ↓ p-PI3K; ↓ p-Akt; ↓ NF-κB	↑ apoptosis	[299]
MCF-7 and MDA-MB-231	1,25(OH)_2_D_3_	↑ Bax and ↑ caspase-3; ↓ Bcl-2	↑ apoptosis	[187]
DU145	1,25(OH)_2_D_3_	↓ CYP24; ↑ VDR	↑ growth inhibition	[300]
LNCaP	HPCD	c-PARP; caspase-3; ↓ EGFR-Akt-GSK-3beta-p70S6k	↑ apoptosis	[301]
DU145	terazosin	↓ Bcl-xl	↑ apoptosis	[302]
PC3	radiation	↓ NF-κB; ↓ cyclin B and/or ↑ p21; ↑ c-PARP	↑ apoptosis	[202]
MCF-7 and MDA-MB-231	X-ray irradiation	↑ Bax; ↑ p73; ↓ Bcl-2	↑ apoptosis	[196]
prostate cancer orthotopic model	radiation		↑ apoptosis	[303]
DU145	ionizing radiation	↓ damage repair	↑ apoptosis	[304]
MCF-7 and MDA-MB-231	hypericin	↓ Bcl-2; ↑ Bax; ↓ p-Akt; ↓ p-Erk1/2	↑ apoptosis	[305]
DU145	tyrphostin AG1024 and X-irradiation		↑ apoptosis	[306]
MCF7-aro and LTEDaro	Exe Exemestane	↑ c-PARP/PARP	↑ apoptosis	[307]
PC3	KSP inhibitor SB715992		↑ apoptosis	[308]
MCF7 and MDA-MB-231	clofarabine	↑ PTEN; ↑ RARB; ↑ CDKN1A	↑ apoptosis	[309]
MDA-MB-231	NP-siCD36	↓ p-p38	↑ growth inhibition	[310]
DU145	survivin RNAi	↑ caspase-3	↑ apoptosis	[311]
PC3	beta-lapachone (bLap)	↓ caspase-3; ↑ NQO1	↑ apoptosis	[312]
LNCaP; C4-2B	daidzein		↑ apoptosis	[313]
MCF-7 cells	equol	↑ Bax/Bcl-xl	↑ apoptosis	[314]
MCF-7	pomegranate extracts		↑ apoptosis	[315]
MDA-MB-231; MCF-7	sulforaphane	↓ KLF4; ↓ HDAC2; ↓ HDAC3	↑ growth inhibition	[316]
MDA-MB-231 and MCF-7	sulforaphane, sodium butyrate	↓ DNMT; ↓ HDAC; ↓ HMT; ↑ HAT methyltransferases and histone methylases	↑ apoptosis	[317]
LNCaP and p53-null PC3	selenite	↑ p21; ↑ Bax	↑ apoptosis	[201]

Bax, BCL2 Associated X; Bcl-2, B-cell lymphoma-2; BIRC7/livin, Baculoviral IAP repeat-containing 7; CDC2, Cyclin Dependent Kinase 2; CDNK1A, cyclin-dependent kinase inhibitor 1A; c-PARP, cleaved-Poly-ADP ribose polymerase; CYP24, Cytochrome P450 Family 24; DNMT, DNA methyltransferase; EGFR, epidermal growth factor receptor; ERα, estrogen receptor α; ERK, extracellular signal-regulated protein kinases; GSK-3β, glycogen synthase kinase-3 beta; HAT, histone acetyltransferase; HDAC, histone deacetylases; Her2/neu, human epidermal growth factor receptor 2; HES1, hairy and enhancer of split-1; HMT, histone methylase; HPCD, 2-hydroxypropyl-beta--hydroxypropyl-beta-cyclodextrin; KLF4, Krüppel-like factor 4; KSP, kinesin spindle protein; NF-κB, nuclear factor kappa-B; NQO1, NAD(P)H quinone oxidoreductase 1; p-Akt, phospho-protein kinase B; PARP, Poly-ADP ribose polymerase; p-Bcl-2, phospho B-cell lymphoma-2; PI3K, phosphoinositide 3-kinase; PTEN, Phosphatase and tensin homolog; p70S6K, p70 ribosomal S6 kinase; RARB, retinoic acid receptor beta; ROS, reactive oxygen species; SLUG/SNAI2,Snail Family Transcriptional Repressor 2; TGFB1I1/ARA55, transforming growth factor beta-1-induced transcript 1 protein; VDR, vitamin D receptor; ↑ = increase; ↓ = decrease.

## Abbreviations

AIF	apoptosis-inducing factor
Akt	protein kinase B
AMPK	AMP-activated protein kinase
ARHI/DIRAS3	Aplasia Ras Homology I
ART	ataxia telangiectasia and Rad3-related kinase
ASK1	apoptosis signaling kinase 1
Bax	BCL2-associated X, apoptosis regulator
Bcl-2	B-cell lymphoma 2
Bim	Bcl-2 Interacting Mediator of cell death
CCNH	cyclin H
CD	cluster of differentiation
cdc	cell division cycle
CDK	cyclin-dependent kinase
CDKN2A	cyclin-dependent kinase inhibitor 2A
CHEK2	checkpoint kinase 2
CHOP	C/EBP homologous protein
CRC	colorectal cancer
CSCs	cancer stem cells
CuZnSOD	copper–zinc superoxide dismutase
CXCL16	C-X-C Motif Chemokine Ligand 16
DNA	deoxyribonucleic acid
DNA-PKcs	DNA kinase catalytic subunit
ECM	extracellular matrix
EGFR	epidermal growth factor receptor
EMT	epithelial–mesenchymal transition
ERs	estrogen receptors
ERK	extracellular signal-related kinase
ESA	epithelial-specific antigen
FADD	FAS-associated protein with death domain
FAKs	focal adhesion kinases
FGF	fibroblast growth factor
FOXM1	Forkhead box protein M1
FSARE	flaxseed aglycone-rich extract FTH1 ferritin heavy chain 1
5-FU	5-fluorouracil
Gli1	GLI Family Zinc Finger 1
GPR30	G protein-coupled receptor 30
GPX	glutathione peroxidase
GSK-3β	glycogen synthase kinase 3-beta
HAT1	histone acetyl transferase 1
HCC	hepatocellular carcinoma
HeLa	human cervical carcinoma cell
HERC5	Hect domain and RLD5
HIF-1α	hypoxia-inducible factor 1α
HOTAIR	HOX Antisense Intergenic RNA
HPCD	2-hydroxpropyl-beta-cyclodextrin
ICAM1	intercellular adhesion molecules-1
IGF-IR	type I insulin growth factor receptor
JAK	Janus kinase
JNK	Jun N-terminal kinase
KCNK9	potassium channel proteins containing two pore-forming P domains
MAPK	mitogen-activated protein kinase
MCM	minichromosome maintenance
MEK	MAP kinase-ERK kinase
miRNAs	microRNA
MMPs	metalloproteases
MnSOD	manganese muperoxide dismutase
mRNA	messenger ribonucleic acid
mTOR	mammalian target of rapamycin
ncRNAs	non-coding RNAs
NFAT1	nuclear factor of activated T cells 1
NF-kB	nuclear factor-kappa B
Notch	signal transducer and activator of transcription
NSCLC	non-small cell lung carcinoma
Oct	octamer-binding transcription factor
OPN	osteopontin
PAI-1	plasminogen activator inhibitor-1
PARP	poly-ADP ribose polymerase
PC	prostate cancer
PCD	programmed cell death
PCNA	proliferating cell nuclear antigen
PDGF	platelet-derived growth factor
PI3K	phosphatidylinositol-3-kinase
PLK-1	polo-like kinase 1
PTEN	phosphatase and tensin homolog
ROS	reactive oxygen species
SARE	soya aglycone-rich extract
SIRT1	sirtuin 1
Snail	Snail homolog 1/2 of drosophila
SHH	Sonic Hedgehog
Sox2	SEX-determining region (SRY) homology box 2
SPI	soy protein isolate
STAT	signal transducer and activator of transcription
TAZ	PDZ-binding motif
TGF-β	transforming growth factor-beta
TNF	tumor necrosis factor
TNFR	tumor necrosis factor receptor
TRAIL	TNF-related apoptosis-inducing ligand
TPA	tetradecanoylphorbol-13-acetate
TSA	trichostatin A
TIMP-1	tissue inhibitor of metalloproteinases 1
Trx	thioredoxin
TSP-1	thrombospondin-1
TWIST	Twist family bHLH transcription factor
ULK1 kinase	UNC51-like kinase-1
uPA	urokinase-type plasminogen activator
VEGF	vascular endothelial growth factor
YAP	Hippo-Yes-associated protein
ZEB1/2	zinc finger E-box binding homeobox 1/2
ZO-1	zonula occludens-1
